# Sustainable One-Pot Production and Scale-Up of the New Platform Chemical Diformylxylose (DFX) from Agricultural Biomass

**DOI:** 10.1021/acssuschemeng.4c03799

**Published:** 2024-08-15

**Authors:** Anastasia O. Komarova, Zezhong John Li, Marie J. Jones, Oliver Erni, Fabien Neuenschwander, Juan D. Medrano-García, Gonzalo Guillén-Gosálbez, François Maréchal, Roger Marti, Jeremy S. Luterbacher

**Affiliations:** † Laboratory of Sustainable and Catalytic Processing, École Polytechnique Fédérale de Lausanne, Station 6, Lausanne 1015, Switzerland; ‡ Industrial Process and Energy Systems Engineering, École Polytechnique Fédérale de Lausanne, EPFL Valais-Wallis, Sion 1950, Switzerland; § Institute ChemTech, Haute école d’ingénierie et d’architecture Fribourg, Boulevard de Pérolles 80, Fribourg 1700, Switzerland; ∥ Department of Chemistry and Applied Biosciences, Institute for Chemical and Bioengineering, ETH Zurich, Zurich 8093, Switzerland

**Keywords:** lignocellulose, carbohydrates, process design, process scale-up, green solvents, platform chemicals, techno-economic assessment, life-cycle analysis

## Abstract

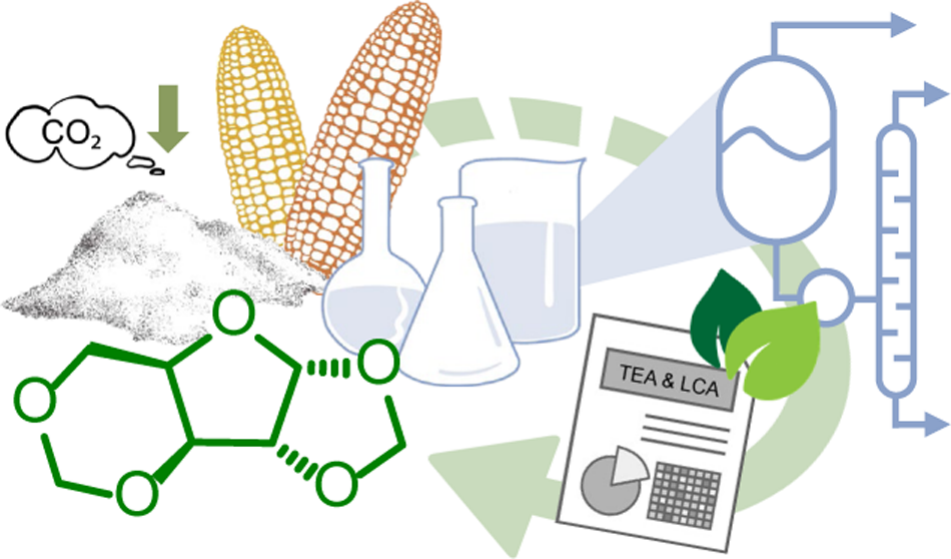

The large-scale production of platform chemicals from biomass requires efficient, cost-effective, and sustainable methods. Here, we present three one-pot synthesis routes for producing diformylxylose (DFX), a sugar-based solvent and platform chemical, using d-xylose or corncobs as feedstocks. With yields of approximately 80%, these routes were seamlessly scaled from lab to kilogram-scale in a 15 L batch reactor. Techno-economic assessment demonstrates the competitiveness of the proposed methods against fossil- and biobased analogues. Life-cycle analysis shows the potential of these processes to reduce environmental and societal impacts from cradle to gate. At the “end of life”, DFX is demonstrated to be inherently biodegradable. Overall, we present a compelling case study of scaling a novel platform chemical guided by techno-economic and environmental concerns leading to balanced cost-competitiveness and life-cycle sustainability.

## Abstract 2

Diformylxylose production from corncobs and isolated xylose was scaled up at high yields in three methods with low cost and life-cycle impacts.

## 1 Introduction

According to a recent assessment, over 99% of the most common chemicals are still not produced sustainably as they transgress at least one of the planetary boundaries.^1^ Meanwhile, there are growing concerns over the sustainability and safety of chemical processes, and an increasing demand to shift away from the reliance on fossil-based products. Biomass can play an important role in this transition since it is the largest source of fixed renewable carbon on earth.

Key to furthering the industrial implementation of biomass conversion is the large-scale production of biobased platform chemicals. Diformylxylose (DFX), a xylose-based diacetal isolated from lignocellulosic biomass via the aldehyde-assisted fractionation technology,^2,3^ demonstrated superior performance as a versatile platform chemical in various applications.^4−7^ For example, DFX was used as a starting material to produce xylitol at much higher yields compared to unmodified xylose.^5^ The use of DFX as an alternative to xylose also doubled the furfural yield in a biphasic water-methyl isobutyl ketone (MIBK) system in the presence of an acid catalyst.^6^ Finally, DFX proved to be a highly effective and nontoxic polar aprotic solvent with comparable performance to toxic and environmentally harmful solvents such as *N*-methyl-2-pyrrolidone (NMP), dimethylacetamide (DMAc), and dimethylformamide (DMF),^4^ whose use in industry has become restricted.^8^ To exploit these and other opportunities, a larger-scale production of DFX is necessary.

A primary objective of DFX scale-up was to identify an abundant and inexpensive source of xylose-rich biomass. Corncobs are a promising feedstock, owing to their high availability and low cost as a major agricultural waste.^9^ An especially attractive feature of corncobs in the context of DFX production is that they contain an important fraction of xylan (20–40 wt %), in addition to the cellulose (30–40 wt %), and a lower amount of lignin (8–15 wt %), especially compared to woody biomass that usually contains 20–30 wt % of lignin and less xylan (typically 5–20 wt %).^3,10,11^ This composition and availability have made corncobs a prime feedstock to produce carbohydrates and especially xylose-based platform chemicals, such as xylitol.^12,13^ A recent study from our group has also demonstrated the potential for corncobs as the feedstock of xylose-based sustainable polyesters.^14^

More generally, notable examples of carbohydrate-based solvents and platform chemicals include 2-methyltetrahydrofuran (2-MeTHF),^15^ γ-valerolactone (GVL),^16^ dihydrolevoglucosenone (Cyrene)^17^ and its ketal derivatives,^18^ tetrahydropyran (THP),^19^ levulinic acid (LA) and its derivatives,^20^ as well as diols and polyols.^21^ The synthesis of these compounds mostly revolves around repeated dehydration and hydrogenation reactions to remove the abundant hydroxyl groups naturally present in carbohydrates. These multiple steps often entail high energy consumption, high cost, and complicated synthetic routes. For instance, GVL and 2-MeTHF can be produced by hydrogenation of sugar dehydration products, furfural or LA, at elevated temperatures (200–300 °C) and pressures (>10 bar).^22,23^ Cyrene can be produced at a high yield (>90%) from the hydrogenation of levoglucosenone, a major product of acid-catalyzed pyrolysis of cellulose in the Furacell process.^17^ A recently introduced biobased ether, THP, is produced via hydrogenation of furfural-derived 3,4-dihydropyran (DHP) at high yield (>98%), but the production of DHP still involves dehydration and hydrogenation of furfural via tetrahydrofurfuryl alcohol.^19^ Production of LA derivatives also suffers from difficult product separation from mineral acids and byproducts, and high reaction temperatures (>200 °C). To reduce the complexity of multistep synthetic routes, one-pot approaches have been proposed recently though they tend to suffer from lower product selectivity compared to the conventional routes.^24,25^ These limitations highlight the need for more cost-effective and sustainable biomass conversion methods.

Here, we report the one-pot production of DFX from d-xylose and corncobs in high yields, while adhering to the principles of green chemistry and OECD guidelines.^26^ New processes were successfully scaled up from lab to multikilogram scale in a 15 L reactor. To assess the viability of DFX production at different scales and cost scenarios, these processes were simulated for techno-economic analysis. We also conducted a preliminary life-cycle analysis (LCA) to evaluate the cradle-to-gate footprint of DFX production. We further performed a biodegradation test to explore the end-of-life of DFX. Overall, this study provides a practical example of how to sustainably transform waste biomass into a valuable platform chemical at low cost and with high efficacy.

## 2 Results and Discussion

### 2.1 DFX from d-Xylose: A Greener Synthesis Design

**Figure 1:**
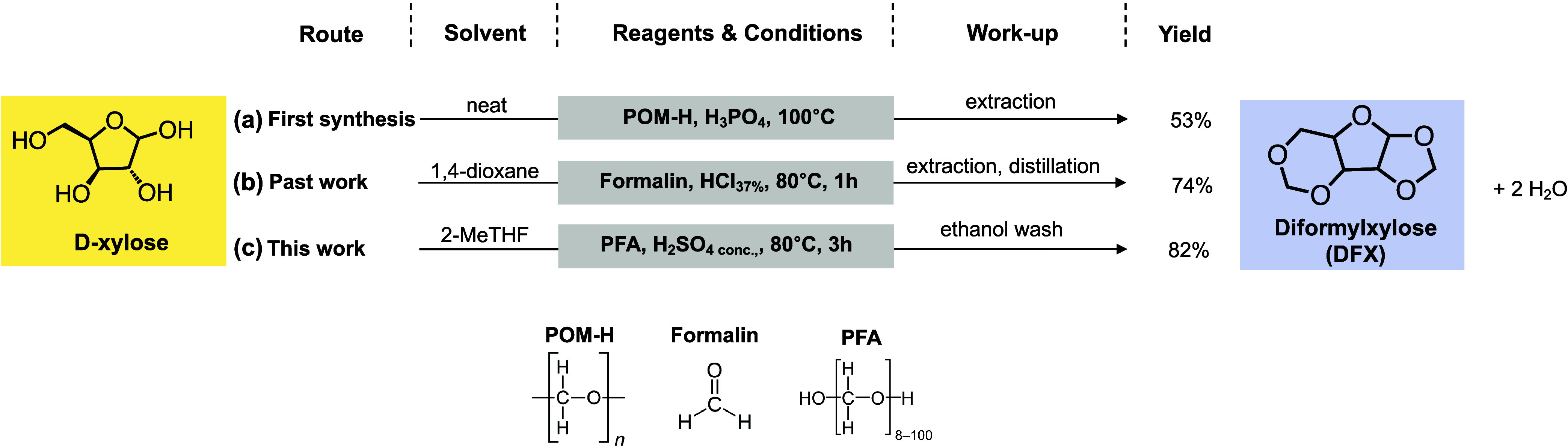
Synthesis routes of DFX using purified d-xylose, acid, and a formaldehyde source. (a) Route for the first reported synthesis of DFX,^27^ (b) route developed by Questell-Santiago et al.,^7^ and (c) the route developed in this work.

Diformylxylose (or 1,2;3,5-*O*-dimethylene-α-d-xylofuranose) was first reported in 1949 by German chemists who reacted d-xylose with polyoxymethylene (POM) in the presence of phosphoric acid, achieving 54% DFX yield (Figure 1, route a).^27^ Our group reported an alternative synthesis procedure using an aqueous solution of formaldehyde (FA) (i.e., formalin) and HCl aqueous solution in 1,4-dioxane, followed by hexane extraction, distillation, and recrystallization to obtain pure DFX in 74% yield (Figure 1, route b).^7^ The use of water-containing reagents (HCl and FA) required a relatively large volume of 1,4-dioxane (1 L per 22 g of d-xylose) to maintain a water content below 10 wt %, thereby preventing the reaction equilibrium from shifting toward reactants. In a recent publication, we replaced the HCl 37 wt % aqueous solution with concentrated H_2_SO_4_ and the formalin solution with paraformaldehyde (PFA).^4^ These modifications allowed us not only to reduce solvent usage 3-fold but also to replace the carcinogenic 1,4-dioxane with a biobased alternative, 2-Me-THF, which is not highly miscible with water. Although PFA, as a formaldehyde-based substance, is classified as a suspected carcinogen under various regulations,^28^ it is widely used in industrial processes,^29,30^ and its associated risks can be mitigated. For example, in our process, we employed a scrubber with 10 wt % sodium bisulfite solution to capture potential formaldehyde emissions, converting them into the nontoxic and biodegradable sodium formaldehyde bisulfite salt. To further minimize risks, we used PFA in the form of beads/pellets to limit dust formation. In addition, PFA is much less toxic by inhalation with a lethal concentration (LC_50_) of 1070 mg/L compared to formalin (LC_50_ = 0.578 mg/L). Also, the oral median lethal dose (LD_50_) for PFA is slightly higher than that for FA (800 vs 500 mg/kg).^31,32^ In this work, we further simplified this synthesis procedure in 2-MeTHF by eliminating the extraction and distillation steps to orient the process toward larger-scale production (Figure 1, route c). Here, DFX crystallizes directly from the concentrated reaction mixture after evaporating the reaction solvent. The resulting crystals have over 98 wt % of DFX after ethanol wash and drying, with an isolated yield of 82%. This simplification saves materials, labor, and energy, which are critical considerations for scale-up. Notably, the direct crystallization of DFX from the concentrated reaction mixture does not occur with the previous methodology employing HCl and formalin even at the same concentration of DFX in the final liquor.

### 2.2 Reaction Sensitivity Analysis Improves Process Scalability

**Figure 2:**
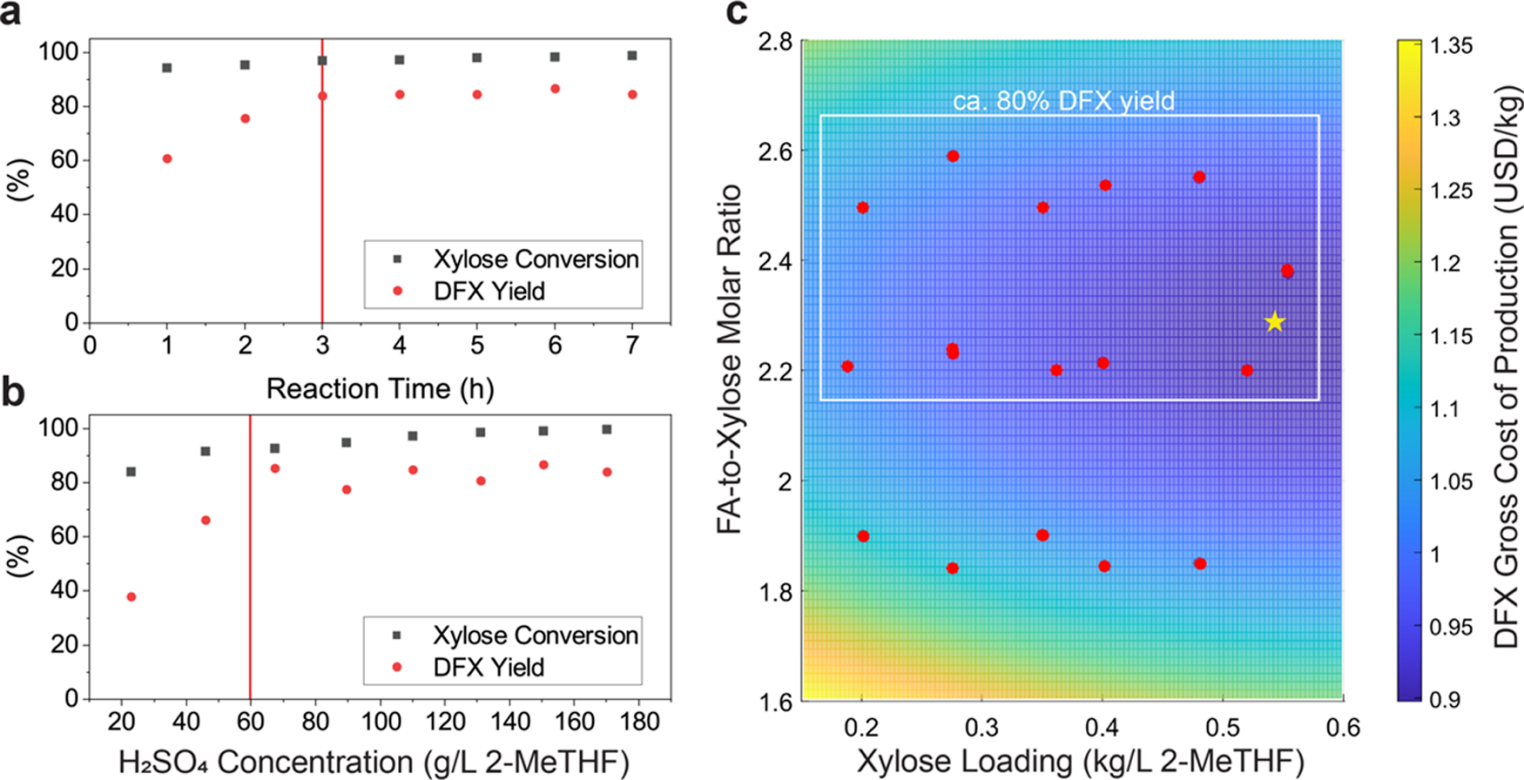
Effect of (a) reaction time (115 g/L xylose and 75 g/L H_2_SO_4_ in 2-MeTHF, FA/xylose mol ratio = 5:1, 80 °C) and (b) H_2_SO_4_ concentrations (50 g/L xylose in 2-MeTHF, FA/xylose mol ratio = 5:1, 80 °C, 3 h) on xylose conversion and DFX yield. (c) Gross cost of production of DFX as a function of xylose loading and FA-to-xylose mole fraction (60 g/L H_2_SO_4_ in 2-MeTHF, 80 °C, 3 h). Data fitted using least-squares regression shown in the color map. The yellow star indicates the lowest cost of production predicted by the regression.

To improve productivity and to reduce the usage of chemicals and process costs, the reaction had to be concentrated. A reaction time of 3 h and acid loading of 60 g/L were fixed to assess the combined effect of xylose loading and formaldehyde-to-xylose molar fraction on gross cost of production (COP) while aiming for a yield of DFX > 80% (Figure 2c). The reaction time and acid loading were selected based on sensitivity analyses (Figure 2a,b). When using PFA work, we calculated the molar equivalence of FA in the mass loading assuming full hydrolysis. Our aggregated objective function was the gross cost of DFX production, which was calculated as the total cost of all raw materials in this experiment divided by the mass of DFX produced (see detailed calculations in Section S2.1, Supporting Information (SI)). Only 1% of the solvent cost was considered in the calculation as we assumed, as a first approximation, that 99% of the solvent could be recycled in an industrial process. The cost of each chemical is summarized in Table S13. Above an FA-to-xylose molar ratio of 2.2, the DFX yield fluctuated around 80% (see Table S1, SI), which agreed with our previous work.^6^ Further increasing the formaldehyde loading did not improve productivity but instead wasted excess formaldehyde, leading to a higher gross cost of production. In contrast, decreasing the formaldehyde-to-xylose molar fraction below ca. 2.2 resulted in greater xylose degradation to humins, as evidenced by the formation of dark insoluble solid during the reaction and consequently, lower yield of DFX (see Table S1, SI).

An empirical model was constructed using a least-squares regression (*R*^2^ = 0.95) to predict the conditions for the lowest gross production cost (indicated by a yellow star in Figure 2c). The regression equation is detailed in Section S2.1, SI, and the optimized conditions were 0.544 kg of xylose and 0.06 kg of H_2_SO_4_ per 1 L of 2-MeTHF, FA/xylose mol ratio = 2.3, at 80 °C for 3 h (Table S1, entry 36). The predicted reaction condition was verified experimentally at laboratory scale (see detailed experimental method in Section 4.2) with a DFX yield of 81% and a gross cost of production of $0.92/kg, which was within 2.2% from the model predictions (see Section S2.1, SI). This condition was then used in the 15 L reactor for the second (final) scale-up test (see Table S1, SI).

The xylose loading did not significantly affect the DFX yield within the tested range. However, at high xylose loading, the reaction mixture became a dense slurry due to the low solubility of xylose in 2-MeTHF at the beginning of the reaction, which made efficient stirring difficult. As the reaction proceeded, xylose was gradually converted to soluble intermediates and eventually to DFX, fully soluble in 2-MeTHF. The formation of water during acetalization further improved xylose solubility. We hypothesize that the reaction initiates at local water enrichments formed around xylose molecules as a similar effect has been observed in systems containing 99 wt % organic solvent and 1 wt % water for fructose dehydration to 5-hydroxymethylfurfural.^33^ Meanwhile, the inorganic acid catalyst is attracted to these water-rich zones, accelerating the reaction. As the reaction proceeds, the produced DFX preferentially moves to the organic bulk while more water is formed, overall ensuring full xylose solubilization and homogenization of the reaction over time. Importantly, introducing water at the beginning of the reaction to solubilize all xylose has limited effectiveness since additional water shifts the equilibrium toward reactants rather than DFX (see Figure S1).

### 2.3 Pilot kg-Scale Production of DFX from d-Xylose and Techno-Economic Considerations

Before the first kg-scale trial in a 15 L reactor, we conducted reaction calorimetry in a 0.5 L-scale (see Section S2.2, SI) to assess possible thermal risks during scale-up (conditions in Table S1, SI, entry 37). We identified two exothermic steps: the addition of sulfuric acid to the reaction mixture (Δ*H* = −60 kJ/mol) and the neutralization of sulfuric acid with aqueous sodium hydroxide (Δ*H* = −126 kJ/mol). At a laboratory scale, the generated heat can be dissipated safely to the surroundings, while at a larger scale, due to the reduced surface-to-volume ratio, precise temperature control is necessary. To ensure process safety, we calculated the adiabatic temperature rise (Δ*T*_ad_) for each exothermic step, which was found to be 52 °C for the acid addition and 39 °C for the neutralization. Additionally, DFX formation was calculated to be exothermic as well (Δ*H*°_rxn_ = −36 kJ/mol, see Section S2.2, SI, for detailed calculation). However, the depolymerization of PFA is a well-known endothermic process, that might compensate for the exothermicity of the former reaction. Overall, we found that the exothermicity during DFX production can be easily controlled with common cooling systems and further mitigated by gradual reagent addition, as no heat accumulation was observed in the calorimetric measurements.

**Figure 3:**
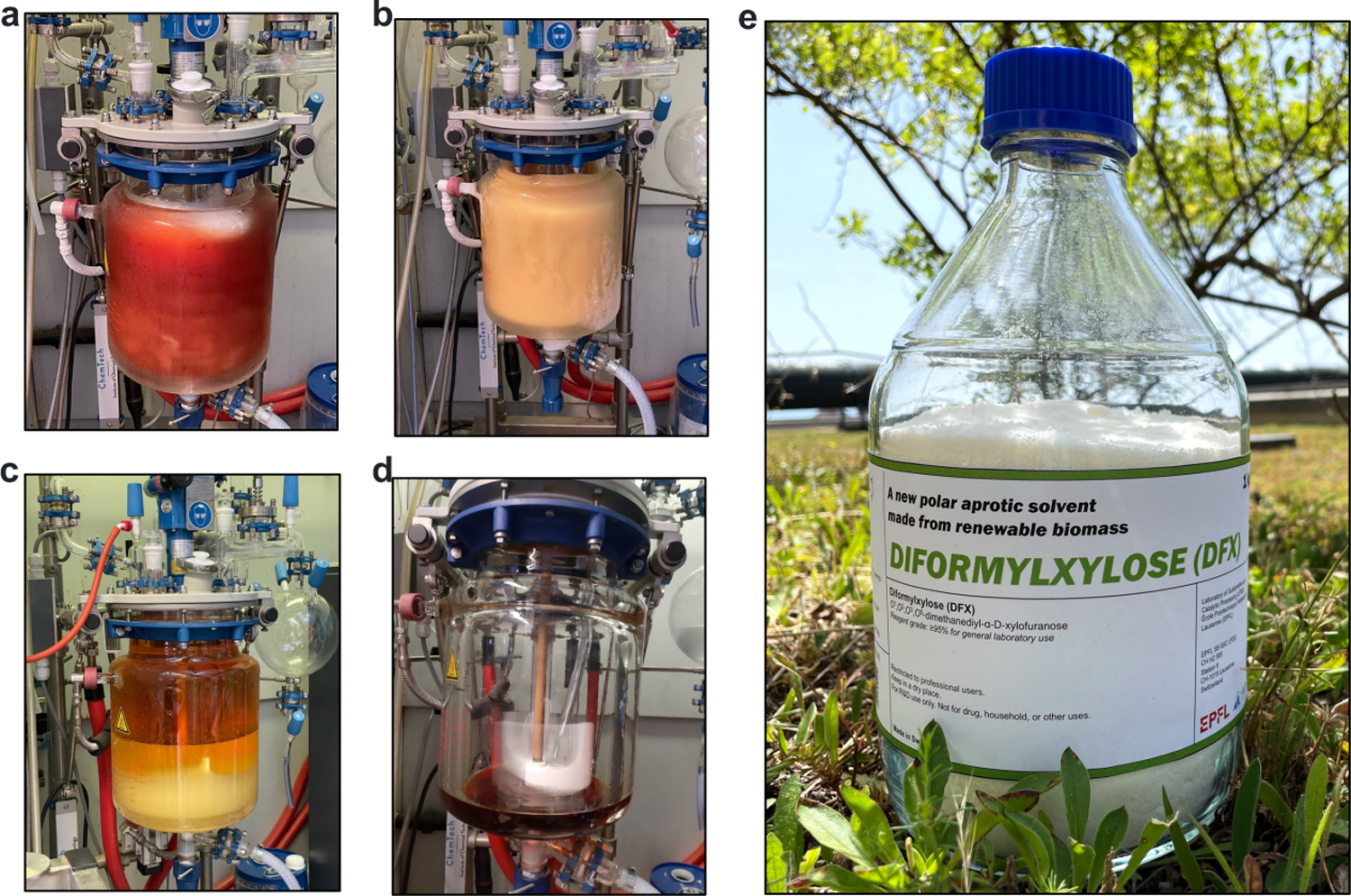
Stages of DFX production in 15 L reactor during the first kg-scale batch. (a) End of the reaction, (b) acid neutralization, (c) phase separation (top: 2-MeTHF, bottom: water and formed Na_2_SO_4_ salt), (d) concentrated organic layer before crystallization, and (e) crystallized pure DFX.

In the first kg-scale batch, the reaction yield of DFX remained comparable to the lab-scale value (see Table S3, SI). However, the isolated yield of the product was only 52% mainly due to mass loss during the workup (Table 1) where a NaOH aqueous solution was used to neutralize the acid, followed by adding extra water to dissolve the resulting Na_2_SO_4_ salt. The large resulting aqueous layer was separated (Figure 3c), leading to DFX loss because of its solubility in water (13 g/100 g at 24 °C).

**Table 1:** Results of kg-Scale Batches Performed in a 15 L Reactor to Isolate DFX from d-Xylose and Corncobs

	xylose route		corncobs route	
	batch 1	batch 2	neutralized	non-neutralized
DFX yields				
DFX reaction yield, mol % on xylose/xylan	73	74	90^a^	90^a^
DFX isolated yield, mol % on xylose/xylan	52	71	76^a^	78^a^
DFX mass losses during workup, wt %	28	3	15	13
DFX isolated yield, wt % of raw biomass	N/A	N/A	19.8^b^	20.3^b^
process green metrics				
*E*-factor	17	1	6	2
process mass intensity (PMI), kg/kg	47	9	13	17
mass productivity, %	2	11	8	6
reaction mass efficiency (RME), %	38	57	49	46
atom economy, %	83	83	N/A	N/A
biomass utilization efficiency stoichiometric (BUE_S_), %	N/A	N/A	97	97
biomass utilization efficiency based on highest molar yield (BUE_H_), %	N/A	N/A	74	76
reaction productivity for DFX, kg/L/h	0.019	0.076	0.031	0.031
process productivity (all products), kg/L/h	N/A	N/A	0.079	0.074
process productivity for DFX, kg/L/h	0.014	0.074	0.026	0.027

a The yields are presented based on the amount of xylose monomer in corncob xylan (see Table S4, SI, for compositional analysis).

b The mass yield is corrected for the mass of added water and formaldehyde to represent the fraction of original biomass that ends up in the final product.

In the second kg-scale trial, we addressed the workup drawbacks to achieve a more than 5-fold increase in productivity compared to the first batch (73 vs 13 g/L/h). Guided by the sensitivity analysis described in the previous section, we increased the xylose loading from 1 to 4 kg and correspondingly increased the PFA loading from 0.6 to 1.8 kg, leading to a higher amount of produced DFX in the same reactor volume at the same reaction yield (73–74%, Table 1). We also reduced the amount of H_2_SO_4_ over 2-fold (0.4 vs 1 kg) compared to the first batch, resulting in a lesser addition of NaOH solution during neutralization (see Table S2 for mass balance). As a result, the mixture remained single-phase after neutralization, and the precipitated Na_2_SO_4_ was removed by filtration, without adding extra water. DFX crystallized directly from a concentrated reaction mixture after evaporation of 2-MeTHF and water. With this workup procedure, the product losses were reduced from 28% for the first batch to only 3% for the second batch (Table 1). Notably, waste Na_2_SO_4_ was reduced over 8-fold and the overall waste generated decreased by 69% compared to the first batch (see Table S2, SI). This improvement was further evidenced by the 17-fold reduction in the *E*-factor, calculated as the ratio of the total mass of waste to the mass of isolated DFX (Table 1). Importantly, the *E*-factor for the second batch reached a value of 1, aligning with the typical range for commodity chemical production.^34^ Overall, we propose that this workup, implying filtration of the salt and its recovery as solid waste, would be easier to implement at an industrial scale than the extraction and distillation steps that would have been needed to minimize product loss in the first batch.

**Figure 4:**
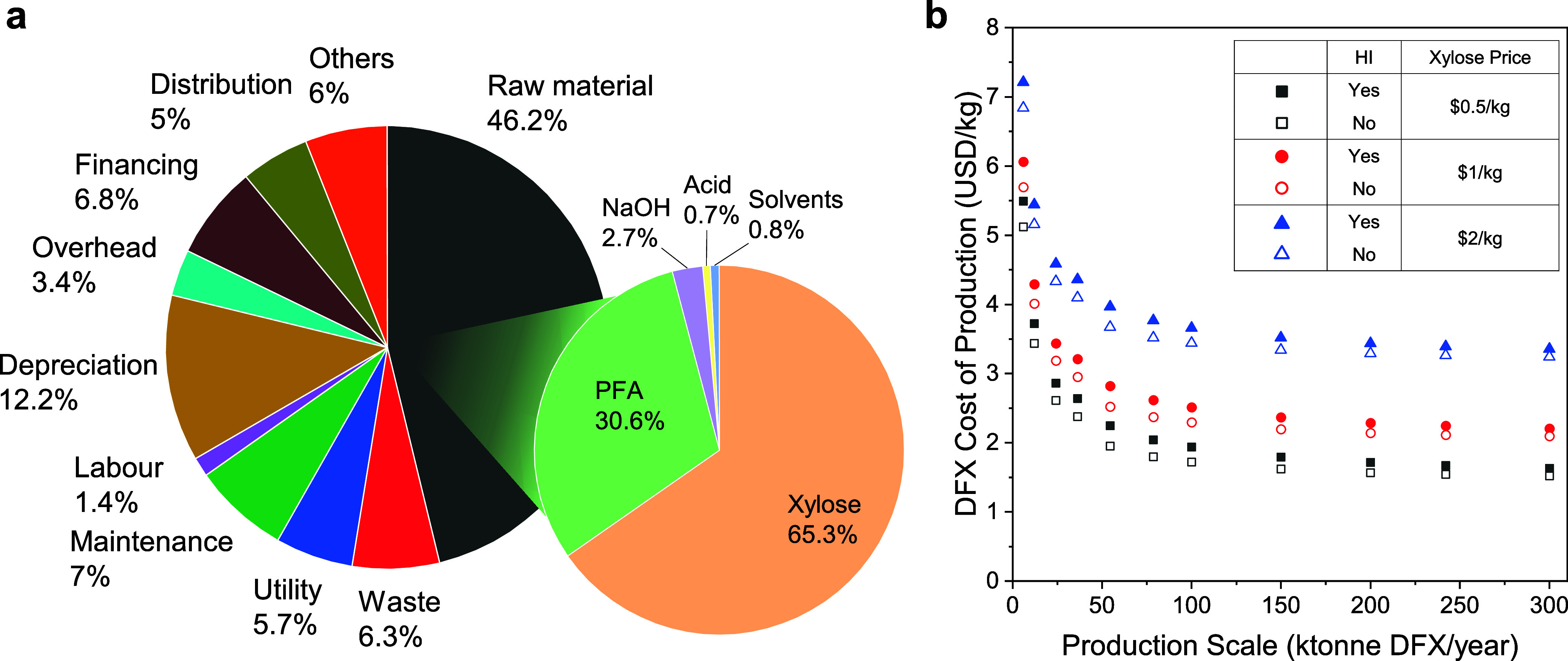
(a) DFX production cost distribution at 150 ktonne DFX/year based on a xylose cost of $0.5/kg, with heat integration. (b) DFX production cost as a function of the production scale at various xylose prices, with and without heat integration (HI).

A preliminary techno-economic assessment of the described process (batch 2 conditions) simulated with Aspen Plus (see Section S4, SI) revealed that over half of the production cost in this scenario is attributed to the cost of raw materials with xylose being the greatest contributor regardless of production scales (Figure 4a). The market price of xylose contains large uncertainties due to a small market size, which could heavily impact DFX production cost (Figure 4b). To accommodate the price disparity of xylose reported in various sources,^35,36^ the DFX production cost was estimated using xylose selling price range of $0.5 to $2/kg. As a result, the DFX minimum selling price varied between $1.5 and $3.4/kg at a production scale of over 100 ktonne DFX/year. Heat integration (HI) was performed to minimize the cost of production (COP) of DFX by varying the minimum approach temperature (see Figures S8 and S9, SI). However, HI did not substantially reduce the production cost due to the process simplicity and low heating and cooling demands (see Figure S10, SI). However, HI reduced utility needs and thus improved the production’s carbon footprint. Overall, the production cost is competitive with other solvents produced at comparable scales (see Table S15, SI). Nevertheless, the strong dependence on one raw material with uncertain pricing motivated switching to a lower-cost agricultural waste biomass as a starting material.

### 2.4 One-Pot Production of DFX from Corncobs

Corncobs as a feedstock for DFX production is particularly interesting since these cobs have a high xylan content of 26 wt % (see Table S4, SI, for compositional analysis). Our group had previously reported the first example of direct synthesis of DFX from woody biomass via FA-assisted fractionation of hardwood at the lab scale, resulting in yields of 76–78 mol % based on xylan content, while isolating FA-stabilized lignin and cellulose pulp.^3^ However, the method presented challenges in terms of scalability due to multiple processing steps, resulting in over 10 h of procedure required to isolate pure DFX from biomass. Additionally, the process involved the use of carcinogenic solvent 1,4-dioxane during pretreatment and the neurotoxic *n*-hexane during the workup. Furthermore, directly applying this method originally developed for hardwood to another species like corncobs led to undesired side reactions, insufficient yields, and chemical waste (see Section S3.1, SI).

Here, we have developed a new and scalable method for processing corncobs, aiming to produce DFX in high yield while retaining other valuable fractions. Similar to the xylose route (Section 2.1), we used solid PFA and the biobased solvent 2-MeTHF for corncob pretreatment. We replaced H_2_SO_4_ with HCl 37 wt % as a catalyst, which allowed for 90 mol % reaction yield of DFX (on a xylan basis) in less than 1 h. In contrast, sulfuric acid required >6 h to achieve the same yield at identical conditions while keeping the same water content of 12 wt % and concentration of [H^+^] = 0.002 mol/g (see Figure S3, SI). The superior performance of HCl could be attributed to chlorine incorporation in the β–O–4 motif of lignin that improves lignin solubility and accelerates biomass deconstruction,^37^ and/or the highly exothermic H_2_SO_4_ and its interactions with water which may lead to undesired local sugar degradation, thereby lowering the DFX yield.

**Figure 5:**
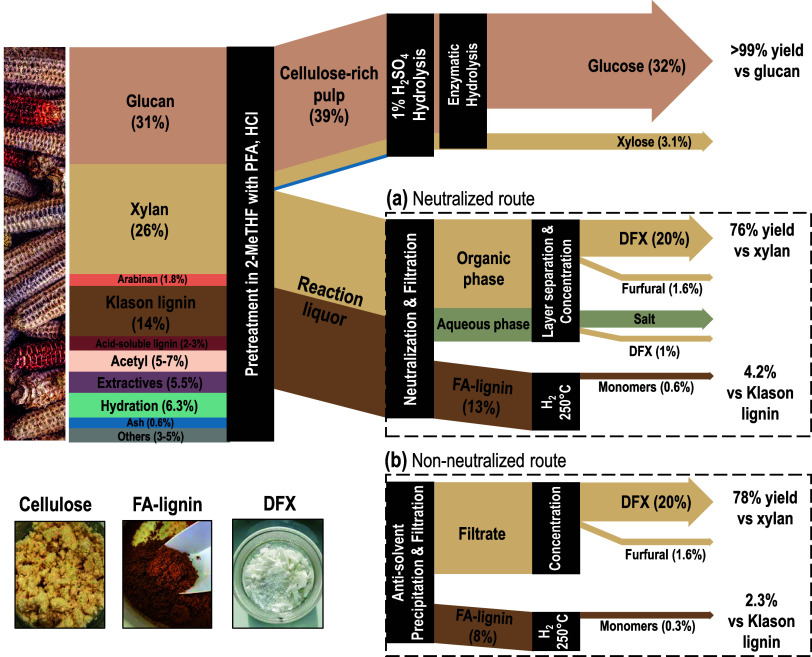
Sankey diagram of the fractionation of corncobs with PFA and HCl depicting the two developed routes and isolated fractions: cellulose, formaldehyde-protected lignin, and formaldehyde-protected xylose (DFX). All of the numbers are provided as weight per weight of nondried and nonextracted biomass percentages with the arrow thickness being proportional to the fraction’s weight. The weight percentages of DFX and FA-lignin have been corrected for the mass of incorporated formaldehyde to match the mass of the precursors as native biomass constituents. The yields represent the results of the kg-scale process.

After the pretreatment, the cellulose-rich pulp was filtered off and enzymatically hydrolyzed to produce glucose (Figure 5). Lignin was then separated from the pulp-free reaction liquor. Following the original procedure developed for hardwood (the neutralized route), the reaction liquor was neutralized and lignin precipitated simultaneously. The lignin precipitate was separated by filtration and the DFX-containing filtrate underwent phase separation. Further distillation of the organic layer resulted in the isolation of DFX with an overall yield of 76 mol % on a xylan basis. However, neutralization posed several disadvantages. (1) The presence of a separate aqueous phase caused DFX losses due to its partial solubility in water. Further extraction could be implemented to combat this, but this adds extra steps to the process. (2) NaCl as the neutralization product must be removed and discarded as additional waste, increasing the environmental burden. (3) Neutralization is an exothermic process that poses thermal safety risks and requires precise cooling control. (4) Neutralization with concentrated NaOH requires extra safety precautions.

To overcome these drawbacks, we developed an alternative strategy (the non-neutralized route), where, instead of neutralization, we added di-*n*-butyl ether as an antisolvent to the reaction liquor to induce lignin precipitation. Di-*n*-butyl ether changed the overall chemical environment of the mixture (e.g., polarity, dispersion forces, hydrogen bonding), destabilizing the lignin–MeTHF interactions and leading to lignin precipitation while keeping DFX, which is soluble in both solvents, in solution. The remaining HCl in the mixture could be removed by evaporation using corrosion-resistant equipment. During this process, the low-boiling azeotrope of water and 2-MeTHF can be distilled first (bp 71 °C). Without the presence of the antisolvent, the acid concentration in the reaction mixture would have continually increased, causing DFX degradation. However, the high-boiling di-*n*-butyl ether (bp 141 °C) remained with the DFX until all HCl was removed, preserving the quality of the product and ensuring a stable and controlled environment during distillation.

The yields of DFX and other fractions isolated in pilot kg-scale process via both routes were consistent with lab-scale results (see Table S6, SI). Overall, 90 mol % of the xylan in corncobs was converted into DFX, resulting in an isolated yield of ca. 20 wt % DFX based on the raw biomass in both routes. The total process productivity for all isolated fractions including cellulose pulp, FA-stabilized lignin, and DFX was comparable to that of the optimized xylose route (Table 1). The individual DFX productivity from corncobs was of course lower than that of the optimized xylose route due to the low xylan density in corncobs by volume compared to the use of isolated sugar.

Table 1 compares other performance metrics for the developed processes (for calculations see Section S1.3, SI). Waste was significantly reduced when switching from the neutralized to the non-neutralized route, as evidenced by the *E*-factor. Reaction mass efficiency (RME) shows the percentage of corncob and PFA mass that remains in the three isolated fractions, indicating atom economy, yield, and reactant stoichiometry. RME remained close to 50% primarily in our processes due to the use of excess PFA, the inclusion of other components in corncobs (26 wt %), and less than 100% yield of isolated products. Biomass utilization efficiency (BUE), is a specialized metric developed for biobased products.^38^ BUE is defined as “the percentage of initial biomass ending up in the end product based on the molar mass of the monomer of the corresponding biopolymer (e.g., xylose from xylan) and target biobased product”. The theoretical stoichiometric BUE (or BUE_S_) (i.e., assuming 100% isolated yield of DFX from xylan) is as high as 97% as only four hydrogens in xylose are substituted to form the final product. Considering the isolated yield of DFX on a xylan basis, the real biomass utilization efficiency (BUE_H_) is 74–76% (Table 1), demonstrating the process’ ability to use natural chemical structures very efficiently.

### 2.5 Techno-Economic Assessment of Corncob Routes

**Figure 6:**
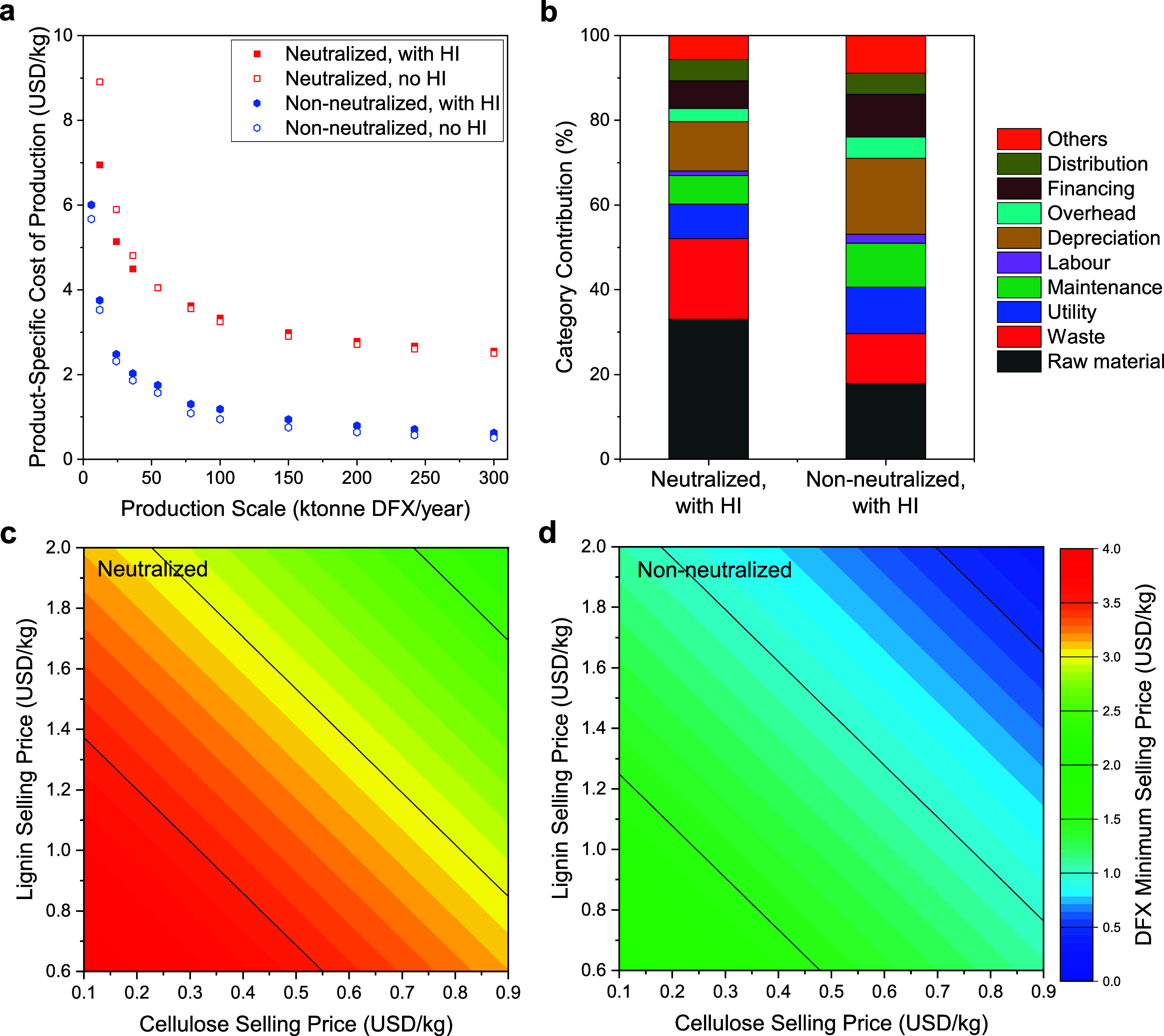
(a) Product-specific cost of production of DFX at various production scales compared between the neutralized and non-neutralized routes with and without heat integration (HI), assuming a selling price of $0.94/kg for lignin and $0.86/kg for cellulose. (b) Cost distribution of the neutralized and non-neutralized route at 150 ktonne DFX/year with heat integration. The minimum selling price of DFX as a function of the lignin and cellulose prices for (c) neutralized and (d) non-neutralized routes with heat integration and at a production scale of 150 ktonne DFX/year.

The economic feasibility of the two corncob-based production routes was assessed with process simulations using the experimental results (Figure 6). Due to the coproduction of lignin and cellulose alongside DFX in these processes, the DFX cost of production (COP) can be calculated by either prescribing selling prices of the other two products (product-specific) or averaging the COP over the total mass of the three products (weight-specific). We focus on the former, which is expected to be more realistic since different products would have prices specific to their market reality (nevertheless, we report the weight-specific prices in the Figures S11 and S12, SI). The non-neutralized route is far more cost-effective over the entire range of production scales. The COP stabilizes below $0.94/kg at a scale above 150 ktonne DFX/year regardless of HI, assuming a selling price of $0.94/kg for Kraft lignin^39^ and $0.86/kg for cellulose.^40^ This price range is much lower than via the xylose route even with the lowest xylose price estimations. In contrast to the other routes requiring constant replenishment of acid and base, the non-neutralized route embodies less material cost (17.9% of the total COP), compared to 46.2% in the xylose route and 33.0% in the neutralized route. The costs associated with wastewater disposal are notedly reduced by eliminating the water loading through acid neutralization and lignin washing. With heat integration, the DFX production cost was reduced in the neutralized route at a production scale below 50 ktonne DFX/year, whereas the price stayed virtually unchanged for a higher production scale. Similar to the xylose route, heat integration slightly increased the COP in the non-neutralized route. This can be attributed to the lesser utility savings compared to the costly heat exchanger network needed for heat integration (see Figure S10, SI). The production of 50–150 ktonne DFX/year would require a corncob supply of 163–490 ktonne/year. Though similar feedstock requirements have been met in the bioethanol production at multiple industrial plants around the United States,^41^ this significant input could also be supplemented by other xylan-containing biomass sources. With an average cob yield of about 200 tonnes/km^2^ in the U.S.,^42^ this translates to 815–2450 km^2^ of farmland, representing 0.2–0.6% of the total U.S. cornfields in 2021.^43^ Constructing the plant close to the corn producing regions would likely be an important factor in managing supply chain uncertainties.

Despite the insensitivity of the DFX production cost to the corncob price (see Figure S8, SI), the process economy heavily depends on the selling prices of cellulose and formaldehyde-protected lignin. Due to the price uncertainty of these two fractions that are from a new process and have unique characteristics, we studied the effect of said prices on the minimum selling price of DFX (Figure 6c,d). For the lignin, we explored a range that varied from the average Kraft lignin price of $0.6/kg^39^ (which is considered to be very low-quality lignin) all the way to $2/kg. For cellulose, we considered a range between $0.86/kg^40^ based on high-quality bleached pulp and $0.1/kg based on a quarter of the minimum glucose selling price to account for additional processing steps.^44^ The selling prices of lignin and cellulose were corrected to the 2021 price level from the prices in the reporting year using the U.S. Consumer Price Index (CPI).^45^ The DFX minimum selling price is $1.87/kg using the non-neutralized route at the lowest lignin and cellulose prices we considered ($0.6/kg for lignin and $0.1/kg for cellulose), which is still reasonable for a biobased solvent (for comparison prices of other common aprotic solvents are given in the Table S15, SI). Such price can be much reduced if the aldehyde-protected lignin is traded at a higher price than low-quality Kraft lignin and cellulose can be used to make more valuable products than glucose.

### 2.6 Comparative Cradle-to-Gate Life-Cycle Analysis

We performed cradle-to-gate life-cycle analyses (LCA) of the three DFX production routes to compare their overall sustainability and environmental impact to that of existing petrochemical solvents (see Section S6 for a detailed description of the methodology).

**Figure 7:**
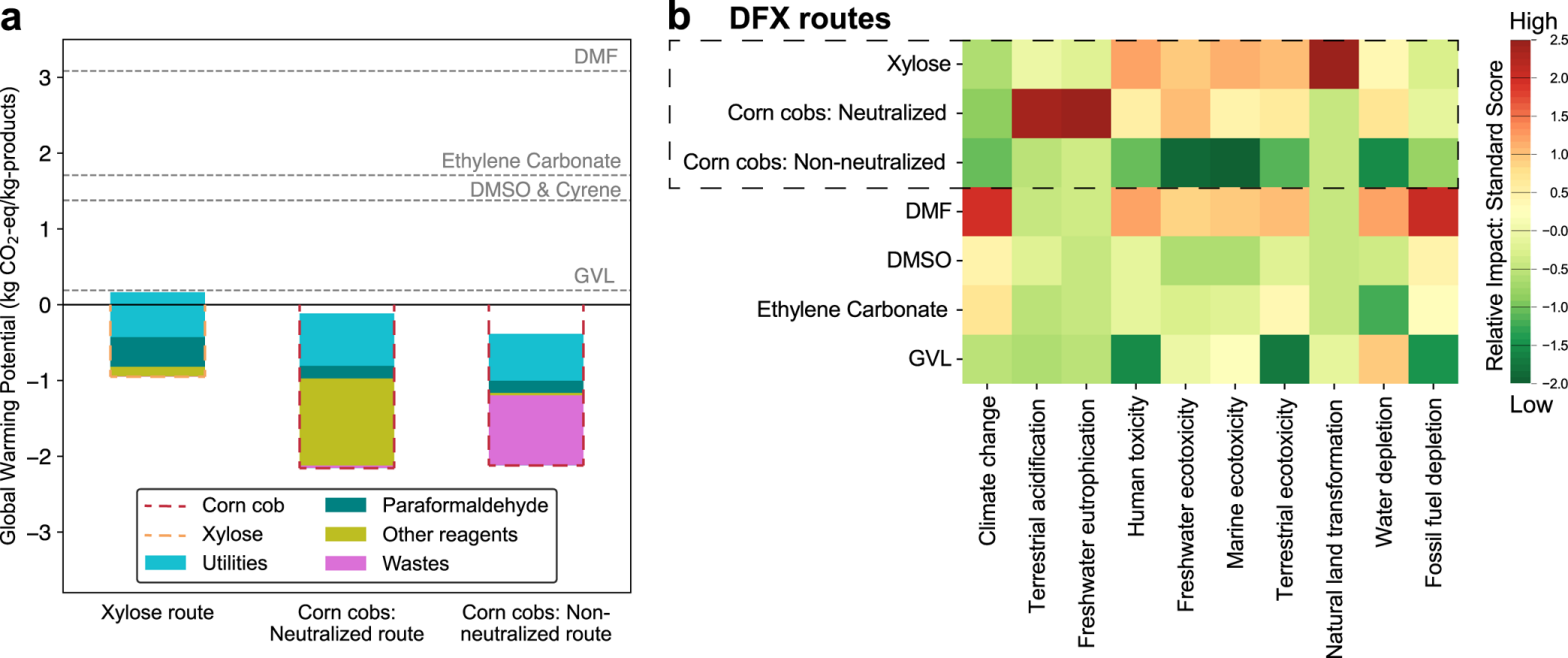
(a) Global warming potential (GWP) impact of DFX for the three process routes. The dashed red and orange lines represent the biogenic carbon of corncobs that is taken up during growth. The top of each bar represents the total GWP value for the corresponding route. Utilities include steam produced using natural gas and cooling water; wastes include the treatment of spent solvents, wastewater, and salts; other reagents include solvents (2-MeTHF, ethanol, and di-*n*-butyl ether), homogeneous acids (HCl or H_2_SO_4_), and the neutralizing agent. (b) Heatmap comparing different DFX production scenarios for 10 midpoint impact categories of the ReCiPe methodology. Data were standardized within their respective categories, and the standard scores were color-mapped (see calculations details in Section 6.4, SI): dark green indicates a significantly lower-than-average impact (more than twice the standard deviation); yellow, average impacts; and dark red, a significantly higher-than-average impact (more than twice the standard deviation). The nonstandardized data are given in the Tables S16 and S19, SI.

The LCA revealed a global warming potential (GWP) impact of 0.16 kg CO_2_ equivalents (kg CO_2_-equiv) per kg of DFX when using xylose as feedstock, which is 93% lower than for dimethyl sulfoxide (DMSO) and Cyrene (Figure 7a), despite the use of fossil-based paraformaldehyde and steam produced from natural gas, which are the major carriers of the environmental impact in this scenario. Production of DFX from corncobs, either through the neutralization or non-neutralized route resulted in a net negative GWP impact of −0.12 and −0.39 kg CO_2_-equiv/kg of products, respectively. In the case of the neutralized route, the main burden comes from the makeup of acid and base which cannot be recycled, while for the non-neutralized route, the treatment of spent organic solvents is the main source of emissions. The carbon emission calculation is limited to the end of the production phase and additional carbon emissions would occur at DFX use and disposal, which would be identical for all three production routes. Overall, all routes to produce DFX offer a substantial reduction in CO_2_ emissions compared to other solvents (see Figure S14, SI).

To provide a more comprehensive environmental assessment, we extended our analysis beyond climate change and included nine additional indicators from the ReCiPe framework (Figure 7b). The production of DFX from xylose can be economically advantageous at a small industrial scale and low xylose price (e.g., 30 ktonne/year, $0.5/kg xylose) among the three routes. However, this route suffers from a high impact on natural land transformation due to the inefficient washing step with biomethanol during xylose purification. The extensive use of NaOH in the neutralized route explains its high impact on acidification and freshwater eutrophication. The non-neutralized route provides a minimal environmental footprint in all impact categories. This route makes the life-cycle impacts of DFX comparable to those of GVL while reducing water usage and production cost (see Table S19, SI). These results demonstrate how the optimization of downstream processing in this case reduces energy and material needs, thereby minimizing the environmental footprint of the process. Once the impact of the process is minimized, the environmental benefits of DFX production are closely tied to the sustainability of the corncob supply chain.

### 2.7 Biodegradability of DFX

The cradle-to-gate LCA of DFX covered its life-cycle stages up to where the product is ready for distribution, and it does not account for disposal. To explore possible end-of-life scenarios for DFX, we performed a biodegradability assessment under aqueous aerobic conditions by manometric respirometry test following OECD 301F guidelines (see Section S7, SI).^46^

DFX showed an overall 47% biodegradation after 28 days of incubation with microorganisms, while d-xylose exhibited near-complete biodegradation, which is consistent with the literature (Figure S15). In comparison, 2-MeTHF provided only 5% biodegradation,^47^ GVL—70–93%,^48^ and dimethyl isosorbide was not biodegradable at all (0%) in the same test.^49^ DFX fit into the category defined by degradation between 20 and 60% within a 28-day window is referred to as “inherently biodegradable” (see Table S20, SI). The presence of a stable acetal group in DFX can possibly make the molecule less accessible to bacteria compared to native sugars. However, the lag phase (time necessary to achieve 10% biodegradation) for DFX was less than 3 days meaning that the typical bacteria from wastewater could adapt to the chemical quite rapidly. The final biodegradation extent was confirmed for both xylose and DFX but using alternative methods: gas chromatography-mass spectrometry (GC-MS) with Soft Ionization by Chemical Reaction Interface Technology (SICRIT) showed 46% degradation of DFX, and high-performance liquid chromatography (HPLC) showed 99.7% degradation of d-xylose in the samples on the 28th incubation day (see Section S7.3, SI). The results of this test show the potential of DFX to be biodegraded in natural and technical conditions (such as wastewater treatment plants) on a relatively short time scale, although higher tiers of tests such as OECD 302 or 303 should be conducted to further investigate environmental effects.

## 3 Conclusions

We present scalable methods to produce the new platform chemical diformylxylose from both d-xylose and agricultural waste biomass while balancing cost and sustainability. The developed one-pot processes were successfully scaled up to a multikilogram scale and proven to be economically competitive at different production scales. The cradle-to-gate life-cycle assessment of different production routes indicated the potential for reduced environmental impacts compared to traditional petroleum- and some biobased solvents. The inherent biodegradability of DFX makes it less likely to cause issues in case of environmental leakage.

The relative ease of batch scalability demonstrated here along with the projected low cost, limited environmental burden, and low-risk toxicological profile of DFX makes it an attractive candidate for broader applications. These promising results can be explained in part by the minimal alternation of the xylose structure when forming DFX. This proof-of-concept further demonstrates that retaining natural structures in biobased products leads to inherent advantages when developing and scaling the production of sustainable chemicals.

## 4 Experimental and Simulation Methods

The following section briefly describes the lab-scale experimental procedures. The methodology for multikilogram-scale synthesis follows the same principal steps but requires reactor-specific actions and extra safety precautions (see Sections S2 and S3, SI). Process simulation, TEA, LCA, and biodegradability methods are detailed in the Section S4–S7, SI, respectively.

### 4.1 Lab-Scale Synthesis of DFX from d-Xylose

d-xylose and paraformaldehyde were added to 2-Me-THF in a 10 mL glass reactor. H_2_SO_4_ (95–97 wt %) was added dropwise with stirring at 400 rpm. The reaction mixture was then heated at 80 °C with stirring. The resulting solution was cooled to room temperature. Specific chemical loadings and conditions are specified in figure captions and Table S1. To isolate DFX, the reaction mixture was neutralized with a saturated NaOH solution, filtered, and concentrated in vacuo using a rotary evaporator with a bath temperature of 45 °C. The final residue crystallized upon cooling to room temperature and was filtered while washing with EtOH to remove impurities and byproducts. The resulting DFX product was a white crystalline solid of ≥98% purity with a reaction yield of 81% and an isolated yield of 74%.

### 4.2 Lab-Scale Isolation of DFX from Corncobs

Ground and sieved (0.45–5 mm) corncobs (5 g) were placed in a 100 mL, thick-walled, glass reactor with an oval poly(tetrafluoroethylene) (PTFE)-coated stir bar. To the reactor sequentially added paraformaldehyde (3 g), 2-Me-THF (20 mL), and HCl_aq_ 37% w/w (5 mL). The reaction mixture was heated to 85 °C with stirring at 600 rpm for 1 h. Then, the reaction mixture was cooled to room temperature, filtered, and washed with 2-Me-THF (10 mL) to separate the cellulose-rich solids from the reaction liquor. The liquor was processed in two routes (A and B below).

#### 4.2.1 Neutralized Route

The liquor was neutralized to pH 6–7 with a concentrated aqueous NaOH solution where lignin simultaneously precipitated. Lignin was filtered and washed with water (5 mL). The organic layer of the filtrate was concentrated in vacuo on a rotary evaporator set at 45 °C to remove 2-MeTHF and traces of water. The resulting yellow oil crystallized at +4 °C upon the addition of DFX seed (∼0.001 g). The crystals were washed with EtOH and dried in a vacuum desiccator, affording DFX as white crystals (≥95% pure by gas chromatography-flame ionization detection (GC-FID)) with an isolated yield of 71% based on the initial xylan content in corncobs.

#### 4.2.2 Non-Neutralized Route

Lignin directly precipitated from the reaction liquor upon adding 90 mL of di-*n*-butyl ether. Lignin was then filtered and washed with di-*n*-butyl ether (15 mL). The filtrate was concentrated on a rotary evaporator set at 60–70 °C gradually increasing vacuum from 400 to 25 mbar to remove 2-MeTHF, water, HCl, and di-*n*-butyl ether sequentially. The resulting brown oil crystallized at +4 °C upon the addition of DFX seed (∼0.001 g). The crystals were washed with EtOH and dried in a vacuum desiccator, affording DFX as white crystals (≥95% pure by GC) with an isolated yield of 71% based on the initial xylan content in corncobs.

### 4.3 Biodegradability Test

Biodegradability tests were performed following the OECD 301F guidelines. Briefly, the biological oxygen demand of each compound was measured over 28 days of incubation with bacteria from seed inoculum (InterLab PolySeed) with oxygen and NaOH pellets replenished every 5 days. Each compound was tested in triplicate. The whole experiment was carried out in duplicate.

## Supporting Information Available

Chemicals, materials, and experimental procedures; process modeling, TEA, and LCA details; and biodegradability test details (PDF)
