# Supplementary material for: Sustainable One-Pot Production and Scale-Up of the New Platform Chemical Diformylxylose (DFX) from Agricultural Biomass
Source: ACS Sustain Chem Eng. 2024 Aug 15;12(34):12879–89. doi: 10.1021/acssuschemeng.4c03799 (PMC11351707; doi:10.1021/acssuschemeng.4c03799)
Supplement: Supplementary file 1 — sc4c03799_si_001.pdf [file sc4c03799_si_001.pdf]

Supporting Information

Sustainable One-Pot Production and Scale-Up of the New Platform Chemical Diformylxylose (DFX) from Agricultural Biomass

Anastasia O. Komarova[a,1], Zezhong John Li[a,1], Marie J. Jones[a,b], Oliver Erni[c], Fabien Neuenschwander[c], Juan D. Medrano-García[d], Gonzalo Guillén-Gosálbez[d], François Maréchal[b], Roger Marti[c], Jeremy S. Luterbacher[a*]

[a]*École Polytechnique Fédérale de Lausanne, Laboratory of Sustainable and Catalytic Processing, Station 6, Lausanne, 1015, Switzerland*
[b]*École Polytechnique Fédérale de Lausanne, Industrial Process and Energy Systems Engineering, EPFL Valais-Wallis, Sion, 1950, Switzerland*
[c]*Haute école d'ingénierie et d'architecture Fribourg, Institute ChemTech, Boulevard de Pérolles 80, Fribourg, 1700, Switzerland*
[d]*ETH Zurich, Institute for Chemical and Bioengineering, Department of Chemistry and Applied Biosciences, 8093 Zurich, Switzerland.*

Corresponding author: jeremy.luterbacher@epfl.ch (Jeremy Luterbacher).

[1] Authors contributed equally.

Number of pages: 36

Number of Figures: 16

Number of Tables: 20

Table of Contents

S1. Chemicals and Methods....................3
S1.1 Chemicals....................3
S1.2 Analytical methods....................3
S1.3 Calculation of yields, productivity, and other metrics....................4
S1.4 Compositional analysis of corn cob feedstock....................4
S1.5 Enzymatic hydrolysis of cellulose-rich pulp....................5
S1.6 Hydrogenolysis of formaldehyde-stabilized lignin....................5
S1.7 Biodegradability test....................5
S2. Synthesis of DFX from D-Xylose....................6
S2.1 Lab-scale synthesis from D-xylose....................6
S2.2. Reaction calorimetry experiments....................8
S2.3. Kg-scale synthesis from D-xylose....................9
S3. DFX Production from Corn Cobs....................12
S3.1 Lab-scale production from corn cobs....................12
S3.2 Kg-scale production from corn cobs....................13
S3.3 Short path distillation (SPD)....................16
S3.4 Enzymatic hydrolysis of cellulose-rich pulp....................17
S3.5 Lignin hydrogenolysis....................17
S4. Aspen Plus Process Simulation....................19
S5. Techno-Economic Assessment....................23
S6. Cradle-to-Gate Life Cycle Analysis (LCA)....................27
S6.1 Methods....................27
S6.2 LCA of corn cobs....................27
S6.3 LCA of xylose....................27
S6.4 LCA of 2-MeTHF....................28
S6.5 Comparison to other solvents....................28
S7. Biodegradability assessment by 301 F test....................31
S7.1 Theoretical oxygen demand (ThOD)....................32
S7.2 Determination of biodegradation %....................32
S7.3 Validation of the BOD results by alternative methods....................33
References....................35

S1. Chemicals and Methods

S1.1 Chemicals

The reagents for the lab-scale procedures were purchased as follows: sulfuric acid (95–97% wt/wt, Supelco), hydrochloric acid (37% wt/wt, Merck), D-xylose (≥99%, Sigma-Aldrich), paraformaldehyde (extra pure granules, Carl Roth), 2-methyltetrahydrofuran (>99%, Solvagreen®, stabilized with 250 ppm BHT, Carl Roth), dibutyl ether (>99%, ACROS Organics), sodium hydroxide (pellets, Reactolab SA), ethyl acetate (Thommen-Furler), tetrahydrofuran (stabilized with 250 ppm BHT, Fisher Chemical,), acetone (technical purity, Thommen-Furler), ethanol (Thommen-Furler), enzyme Cellulases (Novozymes CellicCTec2, Sigma-Aldrich), decane (TCI Europe NV), ruthenium on carbon (extent of labeling: 5% wt/wt Ru, Sigma-Aldrich), trisodium citrate dihydrate (99%, ABCR), citric acid monohydrate (99+%, Acros Organics), tetracycline (≥98%, Sigma-Aldrich), cycloheximide (≥94%, Sigma-Aldrich).

For the pilot-scale procedures, the following chemicals were used: D-xylose (CAS 58-86-6), 2-methyltetrahydrofuran (CAS 96-47-9), sulfuric acid (CAS 7664-93-9), paraformaldehyde (CAS 30525-89-4), sodium hydroxide (CAS 1310-73-2), $Na_2S_2O_5$ (CAS 7681-57-4), hydrochloric acid 37% (wt/wt) (CAS 7647-01-0), di-n-butyl ether (CAS 142-96-1), ethanol (CAS 64-17-5).

All commercial chemicals were used without further purification. Water was purified using a Millipore Milli-Q Advantage A10 water purification system.

S1.2 Analytical methods

High-Performance Liquid Chromatography (HPLC)

The reaction yield of DFX and conversion of D-xylose were determined from the sample taken before neutralization (after pretreatment in corn cob routes). This sample was analyzed by HPLC Agilent Infinity 1260 equipped with a refractive index detector, UV-Vis detector, and an Aminex HPX-87H column (BioRad, USA) at 60°C using 5 mM H2SO4 in water at a flow rate of 0.6 mL min−1 as the mobile phase.

Gas Chromatography combined with Flame-Ionization Detector (GC-FID)

The purity of DFX and monomer yield after hydrogenolysis was determined by GC-FID (Agilent Technologies Gas Chromatography System 7890B) equipped with Flame Ionization Detector and Agilent Technologies HP-5 Column.

Gas Chromatography combined with Mass Spectrometry (GC-MS)

Identification of products and monomers after lignin hydrogenolysis was performed using GC-MS (Agilent Technologies 7890B) with an electron ionization source equipped with an Agilent Technologies HP-5MS Ultra Inert column.

Nuclear Magnetic Resonance (NMR) spectroscopy

NMR spectra were acquired with Bruker Avance III 400 MHz spectrometer with BBFO-plus probe.

S1.3 Calculation of yields, productivity, and other metrics

The isolated yield was calculated by dividing the weight of the dry pure DFX by its molar mass (174.15 g/mol) and the number of moles of xylose.

Product loss percentage was calculated as the mass of DFX produced solely by reaction (derived from reaction yield) divided by the mass of isolated DFX, subtracted from 1, and multiplied by 100%.

Process productivity was calculated as the mass of the isolated product (in kg) divided by the volume of the used reactor (15 L) divided by reaction time (in hours).

Reaction productivity was calculated as the mass of the produced product at the end of the reaction (in kg) divided by the volume of the used reactor (15 L) divided by reaction time (in hours).

Green chemistry metrics were calculated using the following equations described in detail in literature:[1]

$$E\ factor = \frac{Total\ waste\ (kg)}{Isolated\ product\ (kg)}$$

$$Process\ Mass\ Intensity\ (PMI) = \frac{Total\ mass\ of\ all\ materials\ used\ in\ the\ process\ (kg)}{Mass\ of\ isolated\ product\ (kg)}$$

$$Mass\ productivity\ (\%) = \frac{1}{PMI} \times 100\%$$

$$Reaction\ Mass\ Efficiency\ (RME) = \frac{Mass\ of\ isolated\ DFX\ (kg)}{Mass\ of\ xylose\ (or\ corncobs)(kg) + Mass\ of\ PFA\ (kg)}$$

$$Atom\ economy\ (\%) = \frac{Total\ mass\ of\ reactant\ atoms\ (g)}{Total\ mass\ of\ DFX\ atoms\ (g)} \times 100\%$$

Biomass utilization efficiency stoichiometric (BUE_S) and biomass utilization efficiency based on the highest reported yield (BUE_H) were calculated as described in detail in literature.[2] Briefly, since only four hydrogens from the initial xylose molecule (MW 150.1 g/mol) become substituted to form DFX, the BUE_S is 97.3%. BUE_H was calculated by multiplying BUE_S value by the DFX isolated yield on xylan basis.

S1.4 Compositional analysis of corn cob feedstock

Quantification of sugars (glucan, xylan, arabinan galactan, mannan), Klason lignin, hydration, extractives and ashes in grinded corn cobs was performed as described in detail in past work.[3] The results of the compositional analysis are provided in SI, Table S4.

S1.5 Enzymatic hydrolysis of cellulose-rich pulp

Enzymatic hydrolysis of the isolated cellulose-rich pulp was used to determine the potential yield of glucose and xylose and was performed as described in past work.[3] The details and results are provided in SI, Section S3.4.

S1.6 Hydrogenolysis of formaldehyde-stabilized lignin

Depolymerization of the formaldehyde-stabilized lignin isolated from neutralized and non-neutralized fractionation procedure was performed by hydrogenolysis in a Parr reactor as described in details in past work.[3] The details and results are provided in SI, Section S3.5.

S1.7 Biodegradability test

The mineral media used in the biodegradability test was prepared following the OECD 301F guidelines.[4] Briefly, it consisted of 0.6 mM KH_2PO_4, 1.25 mM K_2HPO_4, 1.1 mM $Na_2HPO_4·2H_2O$, 0.1 mM NH_4Cl, 0.2 mM $CaCl_2$, 92 µM $MgSO_4·7H_2O$, 0.9 µM $FeCl_3·6H_2O$, and 2 mg/L N-allylthiourea as nitrification inhibitor. The resulting media had a pH of 7.4.

The inoculum was prepared by mixing the content of one capsule Polyseed (Seed Inoculum, Interlab, USA) with 500 ml of prepared mineral medium. The suspension was aerated for 1 h and then allowed to settle before use.

The test set-up consisted of OxiTop measuring system: vessels with pressure-measuring heads and rubber quivers filled with 3-4 NaOH pellets to absorb CO_2. 164 ml of aerated mineral media, containing inoculum was added to a test vessel with a magnetic stirrer. A test compound was added to the mixture to a final concentration of 100 mg/L as recommended by OECD. Filled vessels were placed in the test incubator set at 21°C with a shaking speed of 400 rpm. Biodegradation measurements were taken daily while refilling oxygen and NaOH pellets every 5 days. Each compound was tested in triplicate. The whole experiment was carried out in duplicate. The calculation details are provided in Section S7.

To confirm the results of the 301F method for DFX, we injected the sample from the final 28th incubation day to GC-MS SICRIT with increased sensitivity equipped with Zb-5ms column three times in splitless mode with the temperature program from 35 to 350°C. The calibration curve for DFX is provided in SI, Figure S14, a. To confirm the result for D-xylose we applied the HPLC method the same as described in Section 3.4. The calibration curve for D-xylose is provided in SI, Figure S14, b.

S2. Synthesis of DFX from D-Xylose

S2.1 Lab-scale synthesis from D-xylose

D-xylose (1.0 g, 0.0067 mol, 1.0 mol. eq.) and paraformaldehyde (0.46 g, equivalent to 0.015 mol of formaldehyde, 2.3 mol. eq.) were added to 2-Me-THF (1.57 g, 1.84 ml) in 10 ml glass reactor. Then, H2SO4 (95-97% (wt/wt), 0.115 g, 0.0011 mol assuming 96% acid, 0.17 mol. eq.) was added dropwise with stirring at 400 rpm to avoid the localized concentration of acid, which can degrade the sugar. The reaction mixture was then heated to 80°C for 3h with stirring. The resulting solution was cooled to room temperature (~23-25°C), neutralized with a saturated aqueous solution of sodium hydroxide, filtered, and concentrated in vacuo using a rotary evaporator with a bath temperature of 45°C. The final residue crystallized directly upon cooling to room temperature and was filtered while washing with EtOH to remove impurities and by-products. The resulting DFX product was a white crystalline solid (≥98% pure by GC-FID, reaction yield 81%, isolated yield 74%). Syntheses conducted at other conditions are specified in Figure 2 and Table S1 followed by the same work-up procedures.

Table S1. Experimental results for DFX synthesis from D-xylose and paraformaldehyde catalysed by H_2SO_4 in 2-MeTHF at 80°C in a 10 ml glass reactor determined by HPLC analysis of samples taken at the end of the reaction:

(a) varying reaction time;

Entry	Reaction time (h)	Xylose (g/L MeTHF)	PFA (mol eq.; g/L MeTHF)	Sulfuric acid (mol eq.; g/L MeTHF)	Xylose conversion (%)	DFX yield (%)
1	1	115	5.0; 115	1; 75	94.1	60.5
2	2	115	5.0; 115	1; 75	95.4	75.5
3	3	115	5.0; 115	1; 75	96.8	83.7
4	4	115	5.0; 115	1; 75	97.1	84.4
5	5	115	5.0; 115	1; 75	97.9	84.3
6	6	115	5.0; 115	1; 75	98.4	86.3
7	7	115	5.0; 115	1; 75	98.9	84.3

(b) varying sulfuric acid concentration;

8	3	50	5; 50	0.5; 22.9	84.0	37.8
9	3	50	5; 50	1; 46.0	91.5	65.9
10	3	50	5; 50	1.5; 67.5	92.7	85.0
11	3	50	5; 50	2; 89.4	94.8	77.3
12	3	50	5; 50	2.5; 110.0	97.2	84.5
13	3	50	5; 50	3; 131.0	98.5	80.6
14	3	50	5; 50	3.5; 150.6	99.0	86.5
15	3	50	5; 50	4; 170.1	99.7	83.7

(c) varying formaldehyde to xylose ratio.

16	3	201	1.9; 76.3	0.45; 60	97.7	69.2
17	3	350	1.9; 133.1	0.26; 60	96.9	68.5
18	3	201	2.5; 100.2	0.45; 60	99.9	79.1
19	3	351	2.5; 175.0	0.26; 60	99.8	79.5
20	3	275	2.2; 123.2	0.33; 60	99.8	77.8
21	3	275	2.2; 123.1	0.33; 60	99.8	82.9
22	3	275	2.2; 122.7	0.33; 60	99.8	80.8
23	3	275	1.8; 101.3	0.33; 60	96.0	64.7
24	3	275	2.6; 142.3	0.33; 60	99.9	79.3
25	3	188	2.2; 83.0	0.48; 60	99.8	77.1
26	3	362	2.2; 159.2	0.25; 60	99.7	78.4
27	3	402	1.8; 148.2	0.23; 60	96.2	64.1

28	3	400	2.2; 177.2	0.23; 60	99.6	80.8
29	3	402	2.5; 204.1	0.23; 60	96.3	79.3
30	3	480	2.6; 245.1	0.19; 60	99.3	85.2
31	3	522	2.2; 228.8	0.17; 60	99.3	82.3
32	3	481	1.8; 177.8	0.19; 60	99.7	70.1
33	3	553	2.4; 263.6	0.16; 60	99.2	79.9
34	3	553	2.4; 263.6	0.16; 60	99.3	81.2
35	3	553	2.4; 263.6	0.16; 60	99.3	84.5
36*	3	544	2.3; 250	0.17; 60	96.2	80.8
37**	3	115	3.0; 69	1.5; 110	98.7	79.1

* conditions used in the second batch of the kg-scale synthesis (see Section 4.2 of the main text).
** conditions used in the first kg-scale synthesis and reaction calorimetry.

Gross production cost calculation

The equation used to calculate the gross cost of DFX production is

$$C = \frac{P_{PFA}m_{PFA} + P_{xylose}m_{xylose} + P_{H_2SO_4}m_{H_2SO_4} + 0.01P_{MTHF}m_{MTHF}}{m_{DFX}}$$

where P_i is the unit cost of each chemical as detailed in Table S13, m_i is the mass of each chemical loaded in the reactor; m_{DFX} is the mass of DFX produced based on the HPLC yield. Based on the data in Table S1(c), the following second-order linear regression was conducted to correlate the calculated gross production cost of DFX with xylose loading and formaldehyde to xylose molar fraction. The cost was calculated based on chemical prices in Table S11 assuming a 99% solvent recycling.

$$C = 4.732 - 3.122F - 0.397X + 0.677F^2 + 0.0986X^2 + 0.0405FX$$

where C is the gross cost of DFX production in USD/kg, F is the FA to xylose molar ratio and X in the xylose loading in kg/L 2-MeTHF. The equation was obtained by minimizing the mean square error. The predicted gross case of DFX production is 0.90 USD/kg at the condition used in the final pilot-scale synthesis.

Effect of addition of water

Addition of a varying amount of water at the beginning of the reaction did not improve the DFX yield even though the reaction was hypothesized to start at the local water enrichments that facilitated the dissolution of xylose (Figure S1). This was due to the additional water shifting equilibrium towards reactants rather than DFX.

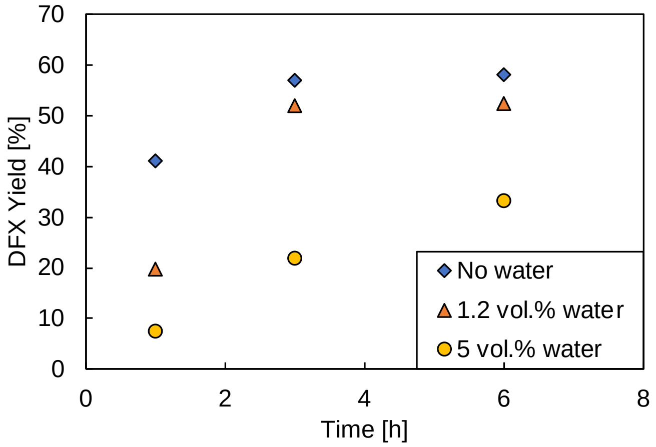

Figure S1 The DFX yield as a function of time with varying addition of water. Reactions conducted with 0.25 g D-xylose, 5 mol. Eq. of PBA and 0.3 g H_2SO_4 in 4mL 2-MeTHF at 110°C in a 10 mL glass reactor.

S2.2. Reaction calorimetry experiments

Heat flow calorimetry, which is a standardized measure in industry, has been used to determine how much heat is generated or consumed during different stages of the process. Enthalpies of reactions during DFX synthesis were determined from heat exchanges measured with a 0.5 L RC1mx reaction calorimeter (Mettler-Toledo, Greifensee, Switzerland) and processed with the software iControl RC1 7.1 (Mettler-Toledo). The reaction was performed at the conditions of the first kg-scale batch (Table S1, entry 37). The exothermic events were determined by the following equation:

$$\Delta_r H = \frac{\int UA * \left(T_r - T_j\right)}{n}$$

where T_r is temperature of the reaction mixture, T_j is temperature of the jacket, UA - heat transfer coefficient of the vessel (determined by the software using the QuickCal method), n – number of moles of sulfuric acid.

The adiabatic temperature difference (ΔT_{ad}) was determined according to the following equation:

$$\Delta T_{ad} = -\frac{\int UA * \left(T_r - T_j\right)}{\sum(m_{solvent,i} * c_{p_{solvent,i}})}$$

where m is the mass, c_p is the heat capacity for all the substances present in the reaction mixture at the start of reaction.

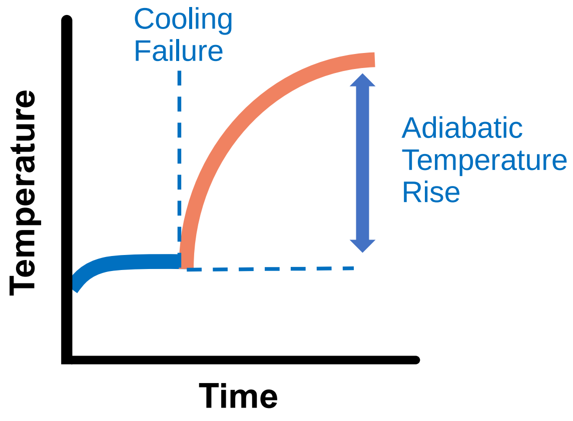

Figure S2. Illustration of the concept of adiabatic temperature rise in the case of cooling failure. The adiabatic temperature rise would be the maximum temperature increase upon a cooling failure assuming no heat exchange with the surroundings.

To simplify the calculations, only the masses and heat capacities of solvents were used. This potentially leads to an overestimation of the adiabatic temperature (i.e. a worst-case scenario).

To calculate the enthalpy of the reaction for DFX synthesis we first determined the enthalpy of formation ($\Delta H^o_{f,\,DFX}$) for DFX. For this, we measured the higher heating value (HHV) of DFX using a bomb calorimeter (IKA C200, Germany). The heat released was corrected to 25°C and 1 atm. The HHV was measured to be -3,443 kJ/mol.

The following DFX combustion equation and standard enthalpies of formation for CO_2 (-393.5 kJ/mol), H_2O (-285.8 kJ/mol), and O_2 (0 kJ/mol), were used to calculate the enthalpy of formation for DFX:

$$C_7O_5H_{10} + 7\ O_2 = 7\ CO_2 + 5\ H_2O$$

$$\Delta H^o_{f,\,DFX} = [7*(-393.5) + 5*(-285.8)] - (-3{,}443) = -740\ \text{kJ/mol}$$

This value of $\Delta H^o_{f,\,DFX}$ was then used together with the enthalpies of formation for D-xylose (-1,058 kJ/mol), formaldehyde (-109 kJ/mol), and water (-285.8 kJ/mol), to calculate the enthalpy of the DFX synthesis reaction:

$$C_5O_5H_{10} + 2\ CH_2O = C_7O_5H_{10} + 2\ H_2O$$

$$\Delta H^o_{rxn} = [2*(-285.8) + 1*(-740)] - [1*(-1{,}058) + 2*(-109)] = -36\ \text{kJ/mol}$$

S2.3. Kg-scale synthesis from D-xylose

Safety information: All operations were done with the usual personal protective equipment (PPE), including goggles, lab coats, and gloves. Additional PPE and the presence of a second person were required for loading 2-MeTHF (gas mask and apron), loading sulfuric acid and sodium hydroxide solution (apron and face shield), loading paraformaldehyde (dust musk or gas mask), and during filtering step (apron and gas mask). Paraformaldehyde was used in the form of beads/pellets to limit dust formation. A scrubber filled with a sodium bisulphite solution 10 wt.% was used for capturing possible formaldehyde emissions, forming sodium

formaldehyde bisulphite, which is not only non-toxic to microorganisms but also a biodegradable substance.

Reactor preparation: A 15 L reactor equipped with an anchor stirrer was put under an inert atmosphere with a flow of nitrogen of 0.3 L/min through the reactor and then connected to a scrubber filled with sodium bisulphite for capturing possible formaldehyde emissions.

Reagent loading: First, 6.31 kg of 2-MeTHF was loaded into the reactor. Then, concentrated sulfuric acid (96 wt.%), 0.444 kg, 4.35 mol, 0.16 mol. eq.) was gradually added to the reaction mixture (approximate flow: 10 ml/min) while keeping the temperature of the reaction mixture below 15°C. Paraformaldehyde (1.76 kg, 58.6 mol, 2.20 mol. eq.) and D-xylose (4.00 kg, 26.6 mol, 1 mol. eq.) were added to the reaction mixture while keeping the inert atmosphere in the reactor.

Reaction: The resulting suspension was heated to 75°C (T_j=85°C) under constant stirring of 200 rpm for 3 hours and then cooled to 10°C. At this point, a sample of the reaction mixture can be taken to determine the reaction yield.

Work-up: For neutralization, a premixed aqueous sodium hydroxide solution (39.3 wt.%, 0.922 kg, 9.06 mol) was gradually added to the reaction mixture (approximative flow: 10-20 mL/min), while keeping the temperature of the reaction mixture below 20°C. The precipitated solid was removed from the bottom of the reactor and filtered with Büchner filter MN 615 (Macherey-Nagel) to remove species of sodium sulphonate, residues of PFA, and non-reacted D-xylose. The filter cake was washed with 2-MeTHF (3 x 1 L) to recover residual DFX from solids and the filtrate was returned to the reactor. The reaction mixture was distilled at 45°C and 225 mbar and then at 80°C and 20 mbar to remove 2-MeTHF and water.

Product purification: The reactor was returned to atmospheric pressure by inserting nitrogen flow and the resulting oil was removed and filtered with Büchner filter MN 615 while washing with EtOH. The filtrated oil was then transferred to a round bottom flask with a stirrer set at 400 rpm. The oil was crystallizing for approximately 2h, then the suspension of crystals was filtered with a vacuum using a Buchner funnel while washing with EtOH (3x0.3L) to achieve the first and the biggest portion of pure DFX crystals (>99% purity by GC-FID). The filtrate was then distilled at a rotary evaporator set at 45°C *in vacuo* to remove excess of EtOH and the crystallization procedure was repeated 2 additional times. Crystals from the three portions were combined and dried in a vacuum oven at 40°C and 100 mbar until constant mass. DFX was obtained as a white crystalline solid (purity ≥99.9% by GC, reaction yield 74%, isolated yield 71%, productivity 0.074 kg/L/h). The reactor was cleaned afterward with acetone and water.

Waste treatment: powder residues (mostly sodium sulphonate) are eliminated as chemical solids; distilled 2-Me-THF and ethanol can be reused; scrubber solution is eliminated as contaminated wastewater.

Analysis: A sample of the reaction mixture is analyzed by HPLC (Section 4.4 of the main text) to calculate reaction yield. The pure product characterized by ^{1}H-NMR in DMSO-d_6: δ 5.97 (d, J = 3.8 Hz, 1H), 4.98 (d, J = 3.7 Hz, 2H), 4.84 (d, J = 6.4 Hz, 1H), 4.63 (d, J = 6.4 Hz, 1H), 4.38 (d, J = 3.7 Hz, 1H), 4.30 (d, J = 2.3 Hz, 1H), 4.03 (d, J = 13.1 Hz, 1H), 3.93 (s, 1H), 3.85 (dd, J = 13.1, 2.1 Hz, 1H).

Table S2. Reagent loadings and mass balance in the DFX production from D-xylose in a 15 L reactor for two pilot-scale batches.

	**Pilot Batch 1**			**Pilot Batch 2**		
**Raw materials**	**Quantity (kg)**	**Mol. eq.**	**wt. % of RX**	**Quantity (kg)**	**Mol. eq.**	**wt. % of RX**
D-Xylose	1.0	1.0	10.0	4.0	1.0	31.9
Paraformaldehyde	0.6	3.0	6.0	1.8	2.2	14.0
H_2SO_4 96% (wt./wt.)	0.98	1.5	9.8	0.44	0.16	3.5
2-MeTHF	7.5	13.0	74.3	6.3	2.7	50.3
**Total RX (kg)**	**10.0**		100	**12.5**		100
**Workup reagents**						
NaOH aqueous	**5.8**			**0.9**		
Water	**2.1**			N/A		
2-MeTHF wash	N/A			**2.3**		
**Total In (kg)**	**17.9**			**15.7**		
**Products**						
DFX end of reaction	0.85	0.73	8.5	3.43	0.74	27.4
DFX isolated	**0.61**	0.53		**3.31**	0.71	
**Recovered**						
2-Me-THF	**6.4**		62.2	**8.6**		50.3
**Waste**						
Aqueous	10.4*			0.7**		
Mother liquor***	0.2			1.1		
Solids (mostly Na_2SO_4 + PFA)	N/A			1.5		
**Total Waste (kg)**	**10.6**			**3.3**		
**Total Out (kg)**	**17.6**			**15.2**		

N/A - not applicable. RX – reaction mixture. "Total In" includes sum of total reaction and total workup reagents. "Total Out" includes sum of isolated DFX, recovered 2-MeTHF, and total waste.

*This aqueous waste contains mostly water and Na_2SO_4 since the salts were aimed to be dissolved and discarded as aqueous layer in this batch.

**This aqueous waste contains mostly water: from NaOH solution and newly formed during the reaction. The solids in the second batch were separated by filtration.

***Mother liquor is leftover after 3rd crystallization and mostly contains humins, reaction intermediates, and non-crystallized DFX.

Table S3. The results of two batches of DFX synthesis from D-xylose performed on lab and kg scales.

**Batch**	**Scale**	**DFX reaction yield (mol %)**	**DFX isolated yield (mol %)**	**Xylose conversion (mol %)**	**Product losses (wt.%)**
1	Vial 10 ml	80	55	99	25
	Reactor 15 L	73	52	99	27
2	Vial 10 ml	81	77	97	5
	Reactor 15 L	74	71	99	3

S3. DFX Production from Corn Cobs

Table S4. Composition of corn cobs measured experimentally using procedure from Amiri et al. and used for the calculation of yields in the fractionation procedures.[3]

Corn cobs content	wt% of the raw biomass
Glucan	30.7
Xylan	25.9
Klason lignin	13.5
Acid soluble lignin	N/M
Hydration	6.3
Extractives*	5.5
Arabinan	1.8
Galactan	N/D
Mannan	N/D
Ash	0.6
Acetyl	N/M
**Total**	**84.3**

N/M – not measured; N/D – not detected.
*Extractives are mainly phenolic compounds such as flavonoid kaempferol and phenolic acids, e.g. protocatechuic acid, p-coumaric acid, caffeic acid, ferulic acid, and vanillic acid.

S3.1 Lab-scale production from corn cobs

To produce and isolate DFX, lignin, and cellulose pulp from corn cobs, we first applied the same conditions as described for birch and beech wood in the past publication.[3] Specifically, ground and sieved corn cobs (4.5 g, 0.45 µm – 5 mm), formaldehyde 37 wt.% aqueous solution (5.2 ml), 1,4-dioxane (25 ml), and HCl 37 wt.% aqueous solution (2.1 ml) were added to the glass reactor together with a PTFE-coated stir bar. The reactor was heated to 95°C with stirring at 400 rpm for 3.5 h. After the pretreatment reaction, the reactor was cooled to room temperature, and 1 ml aliquot was taken and filtered into an HPLC vial to determine the yield of DFX. The yield of DFX was found to be 96% on a xylan basis. The pretreatment liquor was filtered with a Buchner funnel to separate cellulose-rich pulp. The yield of dry pulp after overnight drying in a vacuum oven set at 45°C was 41% vs biomass and >100% vs glucan indicating the presence in the pulp of either traces of lignin and/or degradation by-products. The pretreatment liquor was neutralized with saturated $NaHCO_3$ until pH 7 and concentrated at a rotary evaporator set at 35°C and 60 mbar. A sticky brown solid appeared after evaporation that was not soluble in ethyl acetate. The addition of water to the mixture of the solid and ethyl acetate did not result in lignin precipitation either. Thus, further isolation of DFX was hindered at this point. Therefore, the procedure had to be redesigned and adapted to isolate DFX in high yields as developed and described in this work.

To explore the effect of the reaction time and acid type used in pretreatment on DFX yield, we applied the following conditions: ground and sieved corn cobs (2 g, 0.45 µm – 5 mm), paraformaldehyde (1.2 g), and 2-MeTHF (6.8 g or 8 ml) were added to the glass vial (the same conditions as used for the lab-scale production – section 3.3 of the main text). Then, for the experiment with hydrochloric acid, HCl 37 wt.% aqueous solution (2.4 g, $[H^+]$ = 0.002 mol/g of the mixture) was added to the vial. For the experiment with sulfuric acid, concentrated

H_2SO_4 (95-97 wt.%, 1.4 g, [H^+] = 0.002 mol/g of the mixture) was added to the vial and then 1.5 g of deionized water was added to compensate for the water present in HCl 37% (wt./wt.) and thus, ensure the same water content in both vials (12% wt./wt.). The vials were heated to 85°C with stirring at 400 rpm. The samples were taken after 1h, 2h, 3h, 4h, 5h, and 6h of the pretreatment reaction, filtered into HPLC vial and analyzed by HPLC to determine the reaction yield of DFX (Figure S3).

To explore the effect of the corn cob particle size on DFX yield, we milled corn cobs using a 6-mm screen and then sieved them to separate corn cob samples of three different sizes: 200 µm, 400 µm, and 450 µm - 5 mm. In addition, we tested non-grinded corn cobs of approximately 1-2 cm. We performed a pretreatment reaction of these corn cob samples at 85°C in 2-MeTHF with PFA and HCl 37 wt.% using the conditions mentioned above (or in section 3.3 of the main text). According to the results (Figure S4), the highest yield of DFX could be achieved using the ground corn cob particles of 450 µm - 5 mm size in a 1h pretreatment reaction under used conditions. The particles of this size were then used for the pilot-scale production of DFX.

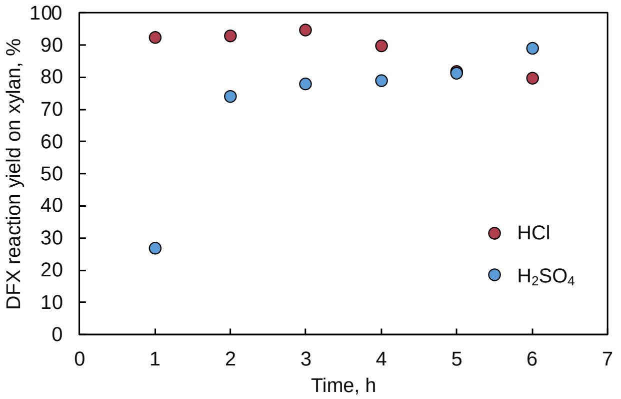

Figure S3. The effect of type of acid and reaction time on DFX pretreatment reaction yield on xylan basis in corn cob pretreatment performed in 2-MeTHF with added PFA at 85°C. The water content of 12% wt./wt. and [H^+] concentration of 0.002 mol/g were kept constant.

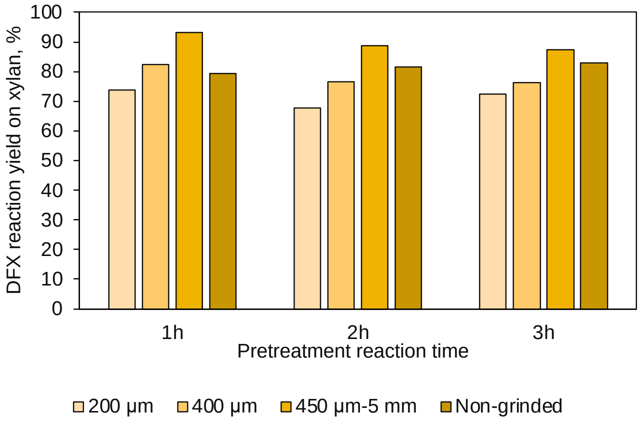

Figure S4. The effect of the size of ground corn cob particles and reaction time on DFX reaction yield after pretreatment in 2-MeTHF with PFA and HCl 37 wt.% at 85°C.

S3.2 Kg-scale production from corn cobs

Safety information: All operations were done with the usual personal protective equipment (PPE), including goggles, lab coats, and gloves. Additional PPE and the presence of a second person were required for loading 2-MeTHF, HCl 37%, di-n-butyl ether (gas mask and apron),

loading of sodium hydroxide solution (apron and face shield), loading paraformaldehyde (dust musk or gas mask), and during the filtering step (apron and gas mask). Paraformaldehyde was used in the form of beads/pellets to limit dust formation. A scrubber filled with a sodium bisulphite solution 10% (wt./wt.) was used for capturing possible formaldehyde emissions. Another scrubber filled with sodium hydroxide solution 10% (wt./wt.) was used for capturing possible HCl emissions.

Reactor preparation: A 15 L-glass reactor equipped with an anchor stirrer was put under an inert atmosphere with a flow of nitrogen of 0.3 L/min through the reactor and then connected through the gas outlet to the first scrubber filled with the aqueous solution of sodium hydroxide (10% wt./wt.) for capturing possible HCl emissions. The first scrubber was connected to the second scrubber filled with an aqueous solution of sodium bisulfite (10% wt./wt.) for capturing possible formaldehyde emissions.

Reagent loading: First, 5.1 kg of 2-MeTHF was loaded into the reactor. Then, aqueous HCl (37% wt./wt., 1.8 kg) was gradually added to the reaction mixture (approximate flow: 10 ml/min) while keeping the temperature of the reaction mixture below 15°C. Paraformaldehyde (0.90 kg) and grinded corn cobs (1.50 kg, 0.45μm<particle size<5mm) were added to the reaction mixture while keeping an inert atmosphere in the reactor.

Pretreatment reaction: The resulting mixture was heated to 75°C (T_j=85°C) under constant stirring of 200 rpm for 1 hour and then cooled to room temperature. At this point, the sample of the reaction mixture can be taken to determine the reaction yield.

Work-up: The reaction mixture was filtered while washing with 2-Me-THF (3x1L) to ensure full separation of the cellulose-rich solids and solubilization of acetalized sugars and lignin. After this, the filtrate was processed separately by two different routes (A and B described below).

***A. Neutralized Route*:** The filtrate was neutralized by gradually adding an aqueous solution of sodium hydroxide (30% wt./wt.) at 0°C (approximate flow: 10-20 ml/min) while keeping the temperature of the reaction mixture below 15°C. The reaction mixture was filtered while washing with water and precipitated lignin was collected as a brown powder. The organic layer was distilled at 45°C and 225 mbar to recover 2-MeTHF and then at 80°C and 20 mbar to concentrate the mixture.

B. Non-neutralized Route: The filtrate was distilled at 45°C and 225 mbar to recover 2-MeTHF until the volume of the reaction mixture reached about 4.0 L. Di-n-butyl ether (9.0 L) was added to the mixture and stirred for 30 min to ensure full precipitation of lignin. The reaction mixture was filtered while washing with di-n-butyl ether and lignin was collected as a pale-yellow powder. The filtrate was distilled at 45°C and 225 mbar to recover 2-MeTHF, remove water and HCl, and finally recover di-n-butyl ether at 80°C and 100 mbar.

Product purification: The reactor was returned to atmospheric pressure by inserting nitrogen flow and the resulting oil was removed. The oil was crystallized at room temperature by adding ethanol (~1/10 of total volume) and seed DFX crystal, followed by filtration and washing with ethanol. The filtrate was distilled to recover ethanol and the purification procedure was repeated 2 more times to isolate all crystal portions. Alternatively, the raw oil can be purified by short-path distillation to isolate pure DFX (section S3.3).

Waste treatment: powder residues (mostly sodium chloride) are eliminated as chemical solids; distilled 2-Me-THF, di-n-butyl ether can be reused; scrubber solutions are eliminated as contaminated wastewater.

Analysis: A sample of the reaction mixture at the end of the pretreatment reaction is by HPLC to determine DFX reaction yield. The pure product characterized by GC-FID and ^{1}H-NMR in DMSO-d_6: δ 5.97 (d, J = 3.8 Hz, 1H), 4.98 (d, J = 3.7 Hz, 2H), 4.84 (d, J = 6.4 Hz, 1H), 4.63 (d, J = 6.4 Hz, 1H), 4.38 (d, J = 3.7 Hz, 1H), 4.30 (d, J = 2.3 Hz, 1H), 4.03 (d, J = 13.1 Hz, 1H), 3.93 (s, 1H), 3.85 (dd, J = 13.1, 2.1 Hz, 1H).

Table S5. Reagent loadings and mass balance in the pilot-scale production of DFX from corn cobs in 15 L reactor.

**I. Pretreatment reaction**		
**Raw materials**	**Quantity (kg)**	**wt % of RX**
Corn cobs	1.5	18.3
Paraformaldehyde	0.9	10.9
HCl 37% (wt./wt.)	1.8	21.5
2-MeTHF	5.1	62.7
**Total RX (kg)**	**9.3**	
DFX mass in the reaction mixture (GC-FID, kg)	0.92	
**II. Cellulose pulp separation**		
**Work-up reagents**		
2-MeTHF wash (kg)	3.0	N/A
**III. Lignin separation**	**Quantity (kg)**	
**Work-up reagents**	**Neutralized route**	**Non-neutralized route**
NaOH aqueous	1.8	N/A
Water (lignin wash)	2.7	N/A
Di-n-butyl ether	N/A	6.9
Di-n-butyl ether (lignin wash)	N/A	1.5
**Total In (kg)**	**16.9**	**20.7**
**Isolated products**	**Quantity (kg)**	
Cellulose-rich pulp (dried)*	0.59	0.59
Lignin (dried)	0.20	0.12
DFX isolated (dried)	0.39	0.40
**Total Isolated (kg)**	**1.18**	**1.11**
**Recovered (kg)**	**Quantity (kg)**	
2-Me-THF + H_2O azeotropic	7.9	9.0
Di-n-butyl ether	N/A	8.4
**Total Recovered (kg)**	**7.9**	**17.4**
**Waste (kg)**	**Quantity(kg)**	
Aqueous	6.9**	1.1***
Organic	0.4	0.4
Mother liquor****	0.2	0.3
**Total Waste (kg)**	**7.2**	**1.7**
**Total Out (kg)**	**16.3 (96%)**	**20.2 (98%)**

N/A - not applicable. RX – reaction mixture.

*Filtered cellulose-rich pulp contained >1kg of moisture together with 2-MeTHF, which were removed by drying and assigned to aqueous and organic wastes, respectively.

** This aqueous waste contains mostly water and NaCl with minor amounts of 2-MeTHF, dissolved DFX, and water-soluble humins.
*** This aqueous waste contains mostly water with dissolved HCl and minor amounts of 2-MeTHF.
**** Mother liquor mostly contains humins, phenolic acids, non-crystallized leftover DFX and minor side products.

Table S6. The results of corn cobs processing on the lab and kg-scale by two routes.

**Workup route**	Scale	DFX reaction yield (mol% vs. xylose)	DFX isolated yield (mol% vs. xylose)	DFX yield (wt.% vs. biomass)	Lignin yield (wt.% vs. Klason)	Lignin yield (wt.% vs. biomass)
**Neutralized**	Vial 30 ml	93	80	19.8	97	13.2
	Reactor 15 L	90	76	19.8	99	13.5
**Non-neutralized**	Via 30 ml	93	71	17.5	69	9.3
	Reactor 15 L	90	78	20.3	59	8.0

The yields on raw biomass are provided for non-dried corn cobs without accounting for moisture.

S3.3 Short path distillation (SPD)

To explore an alternative method for DFX purification in addition to direct crystallization, the raw oil produced from the corn cob batch was subjected to SPD. SPD method enables the separation of low-boiling impurities from high-boiling ones, which could potentially be useful for further utilization of these waste materials. Purification of the raw DFX by SPD method was performed on a short path distillation setup (UIC GmbH, KDL 5 with 0.048 m^2 evaporator surface). In the first run, low-boiling impurities were removed and in the second one – high-boiling impurities. The applied parameters are shown below (Table S7):

Table S7. Selected parameters for SPD of DFX obtained from corn cob processing.

**Parameter**	**1st run**	**2nd run**
Stirring speed / rpm	600	600
Feed flow rate / mL/min	4.0	4.0
Temperature Feed / °C	100	130
Temperature Evaporation Chamber / °C	100	170
Temperature Condenser / °C	80	80
Temperature Residue / °C	100	190
Pressure / mbar	45	45

Table S8. Results of the DFX purification by SPD at kg-scale.

	**DFX, kg**	**Purity, %***	**Impurities, kg**
**Raw oil**	1.18	60-70	
**1st run (low-boiling impurities)**	1.06	85	0.05
**2nd run (heavy impurities)**	0.82	98	0.23
**Losses, kg**	0.07	**Total**	0.28

*The purity was determined by GC-FID. Note that some of the “heavy” impurities cannot be visible by GC-FID due to their low volatility as well as heavy PFA fragments due to their high molecular weight.

S3.4 Enzymatic hydrolysis of cellulose-rich pulp

For enzymatic hydrolysis of the cellulose-rich pulp isolated from corn cobs (section S3.2), we prepared three solutions (citrate buffer 0.1 M with pH of 5, tetracycline solution 10 mg/ml, and cycloheximide solution 10 mg/ml) according to Amiri at al.[3] 0.3 g of pulp was loaded into a 20-ml vial with a PTFE-coated stir bar. To the vial, we added 11.3 ml of the citrate buffer, 0.4 ml of tetracycline solution (to prevent bacterial growth), and 0.3 ml of cycloheximide solution (to inhibit fungi growth). The vial was placed in a shaking incubator (New Brunswick Scientific, Model 126) set at 50 °C and 250 rpm for 1h. Then, 300 µl of cellulase enzyme was added into the vial and the vial was placed back into the incubator for an extra 72 h. After that, the vial was cooled to room temperature and the content was transferred to a 50-ml volumetric flask, washing and diluting with Mili-Q water. 1-ml aliquot was filtered through a filter syringe into an HPLC vial and injected in HPLC twice using the method described in section 3.4 (main text) to determine the yield of glucose and xylose. The experiment was carried out in duplicate on a fresh dry pulp and on a pulp subjected to prior hydrolysis with 1% wt./wt. H_2SO_4 as described below. The result was averaged from two experiments.

Hydrolysis with 1% wt./wt. H_2SO_4: 1 g of the isolated cellulose-rich pulp was placed into a thick-walled glass reactor with PTFE-coated stir bar. To this pulp, we added 12.5 ml of 1% wt./wt. H_2SO_4 aqueous solution prepared by diluting 0.52 g of 95–97% wt./wt. H_2SO_4 with Mili-Q water in a 50-ml volumetric flask. The reactor was heated to 120 ≈ with stirring for 2 h. After that, the reactor was cooled to room temperature and filtered with a Buchner funnel washing twice with acetone and then twice with Mili-Q water. The hydrolyzed pulp was dried in a vacuum oven at 45°C overnight before the enzymatic hydrolysis described above.

Table S9. Results of the enzymatic hydrolysis of the isolated pulp. The values were averaged from two independent experiments on each pulp.

**Sample**	Glucose wt.%			Xylose wt.%		
	vs pulp	vs biomass	vs glucan	vs pulp	vs biomass	vs xylan
**Dry pulp**	38.5	15.2	49.6	3.7	1.5	5.2
**Hydrolyzed dry pulp**	89.3	35.3	99.9	8.9	3.5	12.3

S3.5 Lignin hydrogenolysis

Reductive catalytic fractionation (RCF) of corn cobs: raw grinded and sieved corn cobs (1 g, 0.45 µm – 5 mm) and ruthenium on carbon (5% wt./wt., 200 mg) were added to a 50-mL Parr reactor followed by a bar-type PTFE coated stir-bar and methanol (20 mL). The reactor was then heated to 200°C for 5 h with stirring. The reactor was then cooled to room temperature (~23°C) and depressurized. The n-decane standard (200 µL) was then added to the reaction solution. 1 ml of this solution was filtered into a vial with a 0.2 µm PTFE syringe filter and then injected into the GC-MS for identification and to GC-FID for quantification using programs described in detail here.[3] The appropriate peaks were integrated to determine the lignin monomer yield (Table S11) based on Klason lignin and raw biomass as described in detail here.[3] Yield % of Klason lignin shows the weight percent of produced monomer relative to the total weight of Klason lignin initially present in corn cobs (determined by compositional analysis) and it does not account for the weight of isolated lignin. Yield % of the total raw

biomass shows the weight percent of produced monomer relative to the total weight of non-fractionated raw biomass.

Table S10. Monomer number, structure, molecular weight, effective carbon number (ECN) of the monomers detected during lignin hydrogenolysis of corn cobs.

Monomer number	M0	M1	M2	M3	M4	M5	M6	M7	M8	M25	M26	M9
Monomer structure	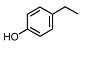	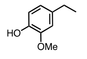	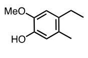	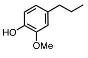	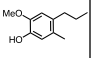	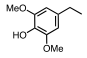	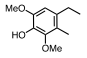	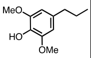	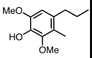	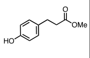	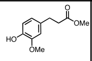	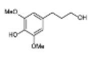
Molecular weight (g/mol)	122.16	152.19	166.22	166.22	180.25	182.22	196.25	196.25	210.27	180.2	210.23	212.24
ECN	7	7	8	8	9	7	8	8	9	10	10	7.5

ECNs were calculated using procedure from Shuai et al.[5] The monomers are listed in the order of their elution time. Monomers M2, M4, M6, M8 can only be produced after formaldehyde-assisted pretreatment due to hydroxymethylation of the guaiacyl and syringyl subunits of lignin. Monomers M25 (methyl 4-hydroxyhydrocinnamate) and M26 (methyl dihydroferulate) are hydrogenolysed and methoxylated derivatives of coumaric acid and ferulic acid that are initially present in corn cob feedstock.

Table S11. Yields of detected lignin monomers from direct hydrogenolysis of the corn cobs.

Monomer	M0	M1	M3	M5	M7	M25	M26	M9	Total
**Yield % (wt/wt) of total biomass**	0.04	0.10	0.15	0.64	0.68	0.99	1.25	0.06	3.92
**Yield % (wt/wt) of Klason lignin**	0.30	0.70	1.13	4.76	5.06	7.33	9.25	0.45	28.98

Yields were corrected for hydration of corn cobs (6.3%). Data produced using procedure from Amiri. et al.[3] M2, M4, M6, M8 monomers are not seen as there is no hydroxymethylation in the absence of formaldehyde.

Hydrogenolysis of isolated lignin: formaldehyde-stabilized lignin isolated from neutralized or non-neutralized fractionation procedure (200 mg) and ruthenium on carbon (5% wt/wt, 100 mg), were added to a 50-mL Parr reactor followed by a bar-type PTFE coated stir-bar and THF (20 mL). The reactor was sealed and then filled with hydrogen gas (40 bar). The reactor was then heated to 250°C for 3 h with stirring and then processed as described above for direct hydrogenolysis procedure. The monomer yields produced from isolated lignin by two routes are shown in Table S12.

Table S12. Yields of detected lignin monomers from the hydrogenolysis of isolated formaldehyde-stabilized lignin.

Lignin produced by route	Monomer	M0	M1	M2	M3	M4	M5	M6	M7	M8	M9	Total
**Neutralized**	**Yield % (wt/wt) of total raw biomass**	0.01	0.07	0.04	0.01	0.02	0.14	0.05	0.13	0.07	0.05	0.59
	**Yield % (wt/wt) of Klason lignin**	0.08	0.44	0.28	0.06	0.16	0.91	0.34	0.87	0.47	0.35	3.96
**Non-neutralized**	**Yield % (wt/wt) of total raw biomass**	N/D	0.02	0.01	0.03	0.05	0.01	0.02	0.16	0.04	N/D	0.32
	**Yield % (wt/wt) of Klason lignin**	N/D	0.21	0.10	0.31	0.64	0.14	0.19	1.95	0.50	N/D	4.06

Yields were corrected for hydration of corn cobs (6.3%). Data produced using procedure from Amiri et al.[3]

S4. Aspen Plus Process Simulation

Aspen plus V11 was employed to calculate the mass and energy balances of continuous processes of DFX production using either pre-isolated D-xylose or corn cobs. The three processes were designed based on the experimental steps in bench-scale and small pilot-scale tests and measured yields as described above. The process flow diagrams are presented in the in Figures S5-7. The non-random two-liquid model (NRTL) was selected as the thermodynamic method. Experimental data from literature was used to obtain more accurate parameters to depict the 2-MeTHF-water interactions.[6–8] The thermodynamic properties of DFX was estimated using the built-in function of Aspen with the measured values of molecular structure, molecular weight, density,[9] melting point,[9] boiling point,[9] and enthalpy of formation calculated in this work. Heat integration was conducted using the build-in Aspen Energy Analyzer with user specified minimum approach temperatures.

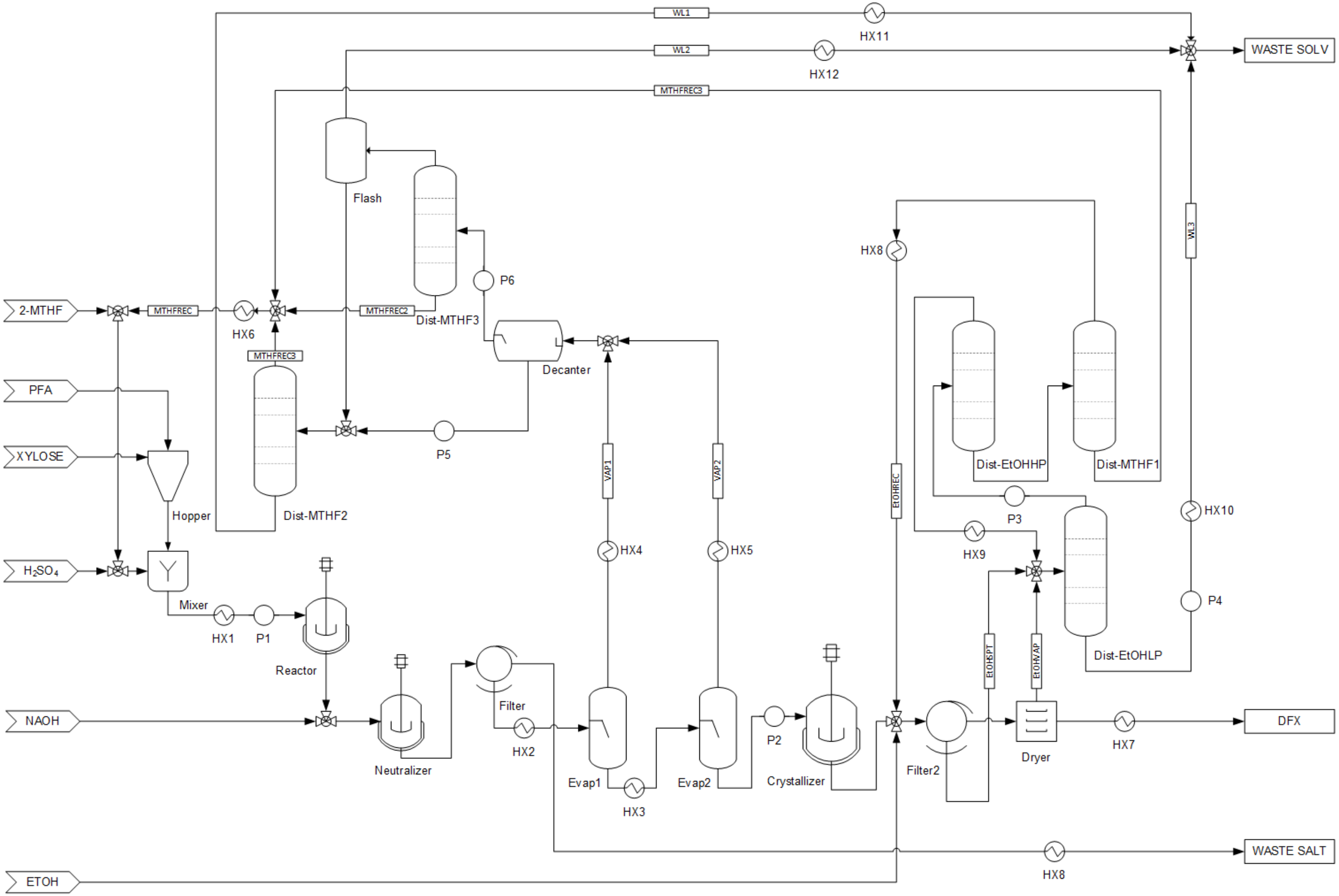

Figure S5. The process flow diagram of DFX production via the xylose route.

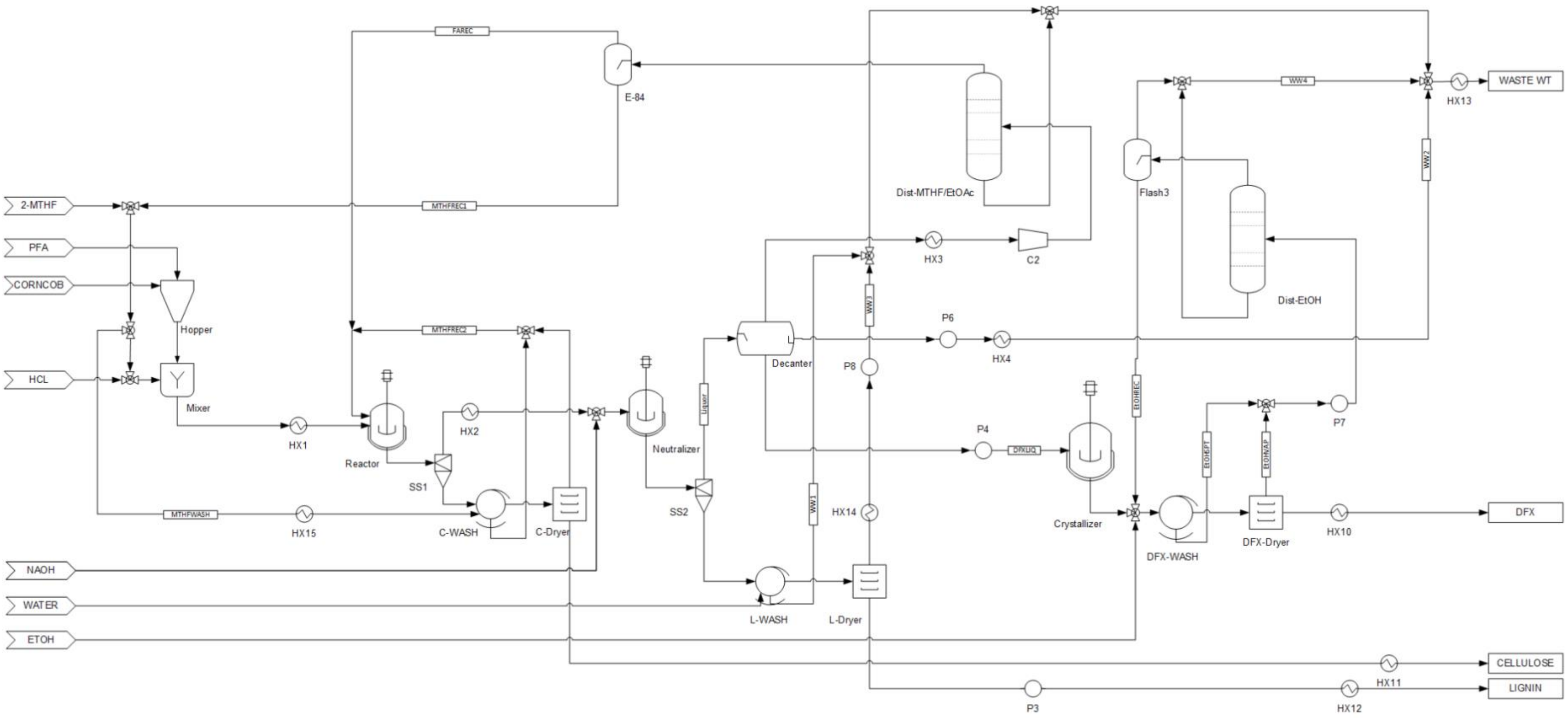

Figure S6. The process flow diagram of DFX production via the neutralized corn cob route.

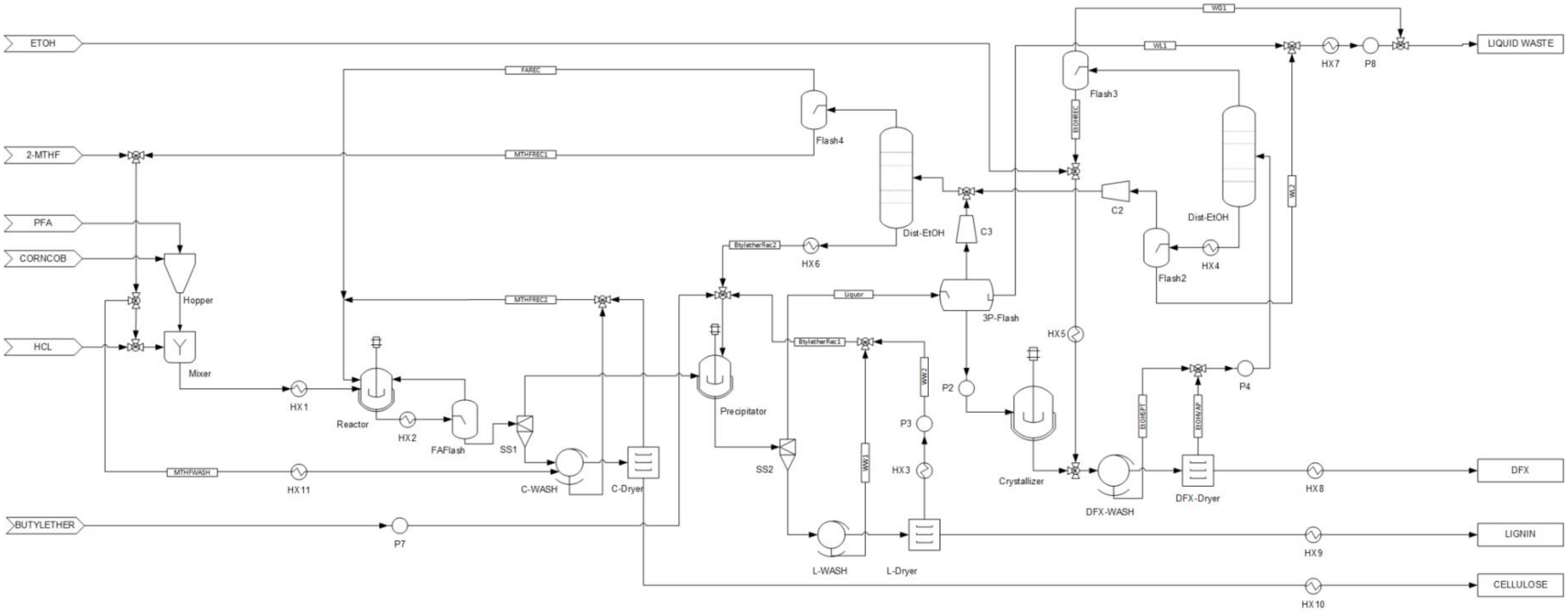

Figure S7. The process flow diagram of DFX production via the non-neutralized corn cob route.

S5. Techno-Economic Assessment

The energy and material balances from the Aspen simulations were used in the techno-economic assessment based on literature pricing indices and correlations.[10,11] The raw material costs, waste disposal charges, and utility costs are summarized in Table S13. All prices were corrected to the 2021 level using the US Consumer Price Index (CPI).[12] The material and energy input as well as the equipment sizing were adjusted for various production scales when calculating costs. The economic factors used to calculate the total capital investment and the total production cost were based on the equations suggested by Ulrich.[13] The plant is assumed to operate for 8000 h per year. The bare module cost of equipment was calculated using correlations suggested by Turton *et al.*[10] and corrected to the price level in 2021 from 2001 using the CEPCI index (708 in 2021 and 374.3 in 2001).[12]

An internal rate of return of 9.16% was used based on the 5-year average of investment return in the chemical manufacturing industry.[14] An annualized equivalence factor was calculated to be 0.103 assuming a 25-year plant life time that converts the initial capital investment to an annualized equivalence. This equivalent annual expense was listed under depreciation in the results.

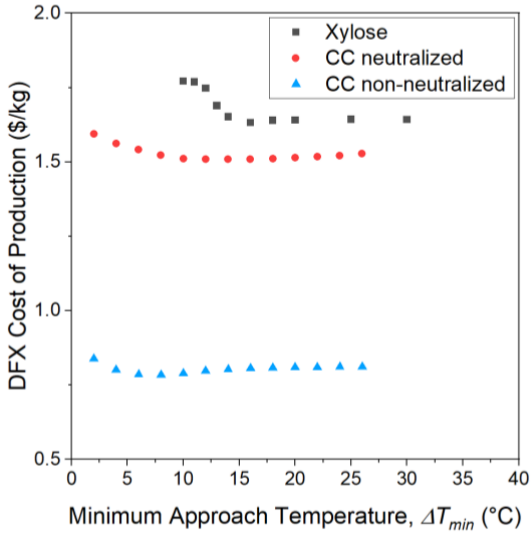

Figure S8. The DFX cost of production as a function of the minimum approach (ΔT_{min}) temperature used in heat integration. The weight-specific price is used for the corn cob (CC) routes. All prices were calculated at the scale of 300 ktonne DFX/year.

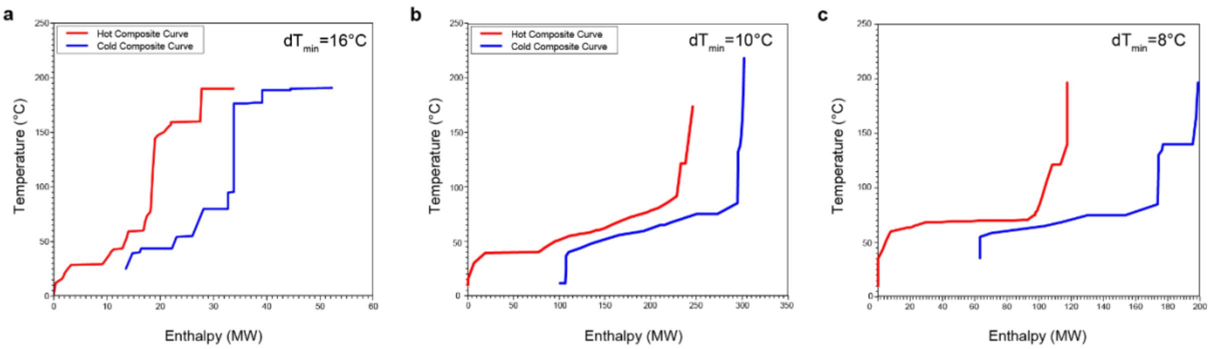

Figure S9. The heat integration composite curves for the (a) xylose, (b) neutralized corn cob and (c) non-neutralized corn cob routes. The minimum approach temperature was selected based on the sensitivity analyses shown in Figure S8.

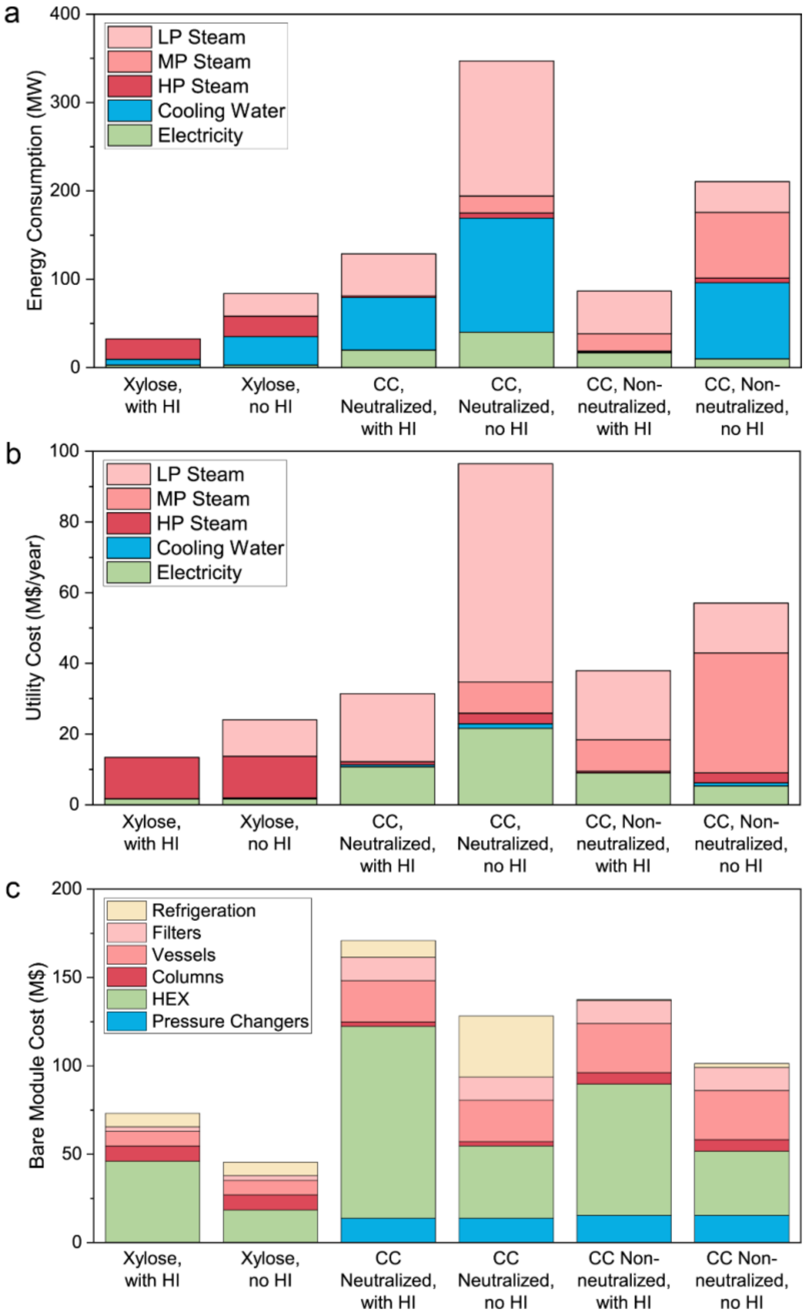

Figure S10. Comparison between with and without heat integration (HI) of the three synthesis routes on (a) energy consumption, (b) utility cost and (c) bare module cost of each equipment category. All calculations are based on the production of 150 ktonne DFX/year. CC stands for corn cob.

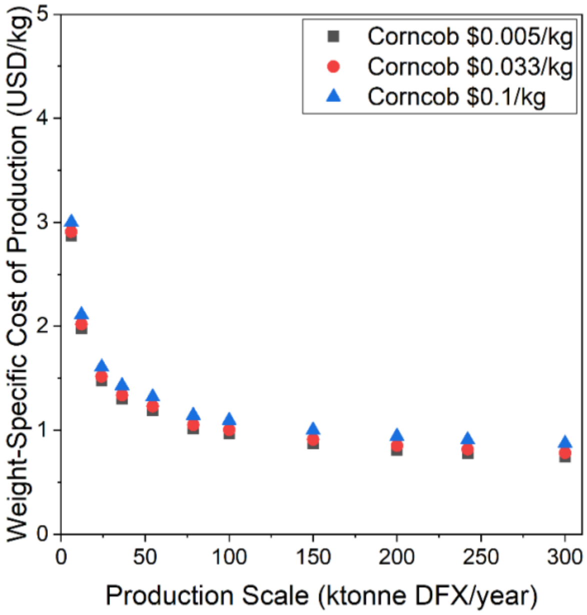

Figure S11. The weight-specific cost of production of DFX at various production scales compared between different corn cob prices. Data are presented from the non-neutralized route with heat integration.

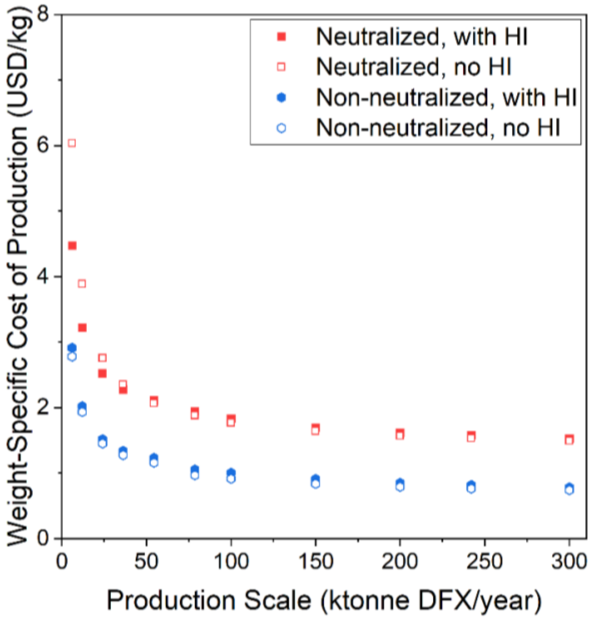

Figure S12. The weight-specific cost of production of DFX at various production scales compared between the neutralized and the non-neutralized routes with and without heat integration (HI) where corn cob price was set at $0.033/kg.

Table S13. Prices of feed and waste streams used in the techno-economic analysis unless specified otherwise.

	Price $/kg	References
**Feeds**		
Xylose	0.5-2	[15,16]
Corn cobs	0.005-0.1	[17]
Paraformaldehyde	0.711	ChemAnalyst*
2-MeTHF	1.77	[18]
H2SO4	0.05	[19], ChemAnalyst*
HCl 37% wt./wt.	0.10	ChemAnalyst*
NaOH	0.26	ChemAnalyst*
Water	0.0001	[20]
Ethanol	0.59	[21], ChemAnalyst*
Ethyl acetate	0.95	[22]
Dibutyl ether	1.72	Alibaba.com
**Wastes**		
Organic liquid	0.23	[23]
Waste water	0.08	[20]
Salt	0.023	[20,24]

* Data retrieved on https://www.chemanalyst.com/Pricing-data, values taken from Q4 2023.

Table S14. Prices of utilities used in the techno-economic analysis.

	Price	References
Electricity	0.0674 $/kWh	[25]
Cooling water	0.354 $/GJ	[10]
HP steam	17.7 $/GJ	[10]
MP steam	15.88 $/GJ	[10]
LP steam	14.05 $/GJ	[10]

Table S15. Production scales and market prices for the common organic solvents.

Solvent	Production scale, ktonne/year	Market prices, USD/kg	References
Cyrene	<10	3-5	[26]
GVL	N/A	1.5-3	[18]
2-MeTHF	25-50	0.5-3	[18]
1,4-dioxane	50-80	1-2	[27]
THF	N/A	0.9-1.4	[27]

N/A – not available.

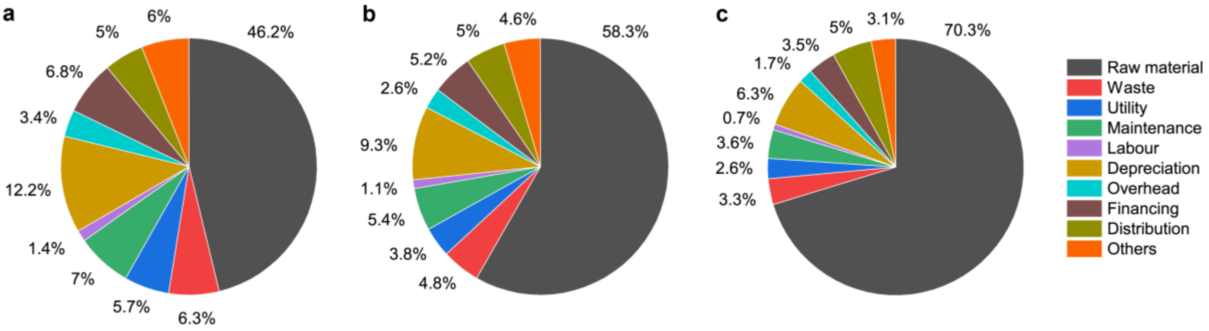

Figure S13. Comparison of DFX production cost at three price scenarios of xylose at **(a)** $0.5/kg, **(b)** $1/kg, and **(c)** $2/kg. The production scale was set at 150 ktonne DFX/year with heat integration.

S6. Cradle-to-Gate Life Cycle Analysis (LCA)

S6.1 Methods

The environmental analysis of three developed DFX production routes was carried out following the ISO 14040/44 standards. We selected 1 kg of DFX as the function for the xylose route and 1 kg of products (DFX, lignin, and cellulose) for the corncob routes. We adopted a cradle-to-gate scope following a cut-off attributional approach. The background system was modeled with the Ecoinvent v3.9.1 database[28] using the Python extension Brightway2,[29] including all upstream activities.

In this preliminary study, the main indicator to measure and compare the environmental impact of DFX was the 100-year global warming potential (GWP 100a) of the chemicals and the process. Nevertheless, we expanded our analysis to nine other midpoints of the ReCiPe framework (2016 hierarchist version)[30] (Table S19). The impacts of the different inputs are listed in Table S16.

The life cycle inventory (LCI) of the process was built from the material and energy flow results of the Aspen Plus simulations with heat integration (Table S13). The following assumptions have been made:

- Life-cycle analysis for di-n-butyl ether is not readily available and was estimated from the Ecoinvent entries for diethyl ether: we substituted the ethanol needed for diethyl ether production with 1-butanol [31] (on a molar basis) and assumed the other reagent and energy needs scaled linearly.
- We assumed the use of bio-based ethanol and methanol.
- We considered the usage of an electricity mix based on renewable energy sources (Swiss energy mix in 2020, 98.4% hydro, 1.1% bioenergy and 0.5% wind).

The following sections will detail our strategies to fill data gaps for environmental impacts related to corn cobs, xylose and 2-methyltetrahydrofuran (2-MeTHF), which is used as the pretreatment solvent.

S6.2 LCA of corn cobs

Corn cobs are residues from maize production and are generally left on the field after the harvest.[32] They are either burned or left to decompose, thus releasing immediately the CO_2 absorbed through photosynthesis. Since corn cobs can be treated as an inevitable waste of maize production, we can assume that all the environmental burdens are carried by the main product (corn grains). The maximum biogenic CO_2 uptake is calculated based on the average carbon content of corn cobs (obtained by ultimate analysis)[33] and corrected for the moisture content (6.6% wt./wt.).[34]

$$m_{CO_2 uptake} = \frac{Carbon\ content}{(1 - Moisture\ content) * 12} * 44 = 1.63\ kg\ biogenic\ CO_2/kg\ corn\ cob$$

S6.3 LCA of xylose

The production of xylose from corn cobs was simulated in Aspen Plus v12 and is based on the patent by Jaffe et al.[35] First, 60 kg/h of biomass (corn cobs) is mixed with 7.62 kg/h of 98.3% (wt./wt.) H_2SO_4 and 513.39 kg/h of water. The resulting stream is heated up to 120 ºC and

enters the reactor at atmospheric pressure. Here, the xylan contained in the biomass is assumed to be 100% hydrolyzed into xylose. The products of the reactor are mixed with 92.97 kg/h of water heated up to 45 ºC and sent to a vacuum flash unit at 0.05 bar. Here, most of the water is removed as vapor and after a small 0.1% purge is recycled back and mixed with the effluent of the reactor. The water is treated with NaOH to neutralize the acid and considered wastewater. The liquid product of the flash is recompressed back to 1 bar and washed with 16.97 kg/h of methanol at 0 ºC. All water and xylose are assumed to be recovered along with 10% of the original methanol wash. The wash is recycled back after a 0.1% purge while the xylose/methanol/water stream is again vacuum flashed at 35 ºC and 0.025 bar. The liquid product, containing 22.42 kg/h of 96% (wt./wt.) xylose, is compressed back to 1 bar, while the vapor is purged. The purged streams are collected and combusted with air, cooled down to 30 ºC, and after a flash separation, the wastewater is removed from the flue gas, which is released to the atmosphere. The LCI can be found in Table S17.

S6.4 LCA of 2-MeTHF

2-methyltetrahydrofuran (2-MeTHF) is a bio-based solvent that can either be produced via furfural or via levulinic acid route.[6] Two published LCA studies on 2-MeTHF production from corn cobs show significant disparities (0.19 kg CO_2/kg for the study by Slater et al.[36],and 5.62 kg CO_2/kg for the study by Khoo et al.) due to different assumptions. Since it was not possible to back-calculate their undisclosed hypothesis from their results, we decided to model 2-MeTHF production from sugarcane bagasse using Aspen Plus v11. Sugarcane bagasse is first fractionated with steam explosion (205 ºC and 30 bar for 13.5 min)[37] and the hemicellulosic fraction is converted into furfural via hydrolysis. The two-step hydrogenation of furfural to 2-MeTHF was modelled based on the work of Leal Silva et al.[38] The biomass pretreatment also yields cellulose which is treated as a by-product that could be sold. The by-product is accounted similarly to cellulose from the DFX process, with mass allocation. The mass and energy balance (with heat integration) of the process are reported in Table S14. The heat requirements are fulfilled by burning additional sugarcane bagasse, taking Lower Heating Value (LHV) of 17 MJ/kg[39] and a boiler efficiency of 80%.[40] The environmental impact of sugarcane bagasse is retrieved from the Ecoinvent database as the waste of sugar cane processing to produce bioethanol. The biogenic CO_2 is thus not accounted for, and burning sugar cane bagasse does not release fossil CO_2. The same reasoning can be extended to the burning of spent 2-MeTHF in the DFX process.

S6.5 Comparison to other solvents

The impact of producing DFX was compared with other commonly used solvents: DMF, cyrene, DMSO, ethylene carbonate, 1,4-dioxane, NMP, DMAc, and gamma-valerolactone (GVL). For the latter, a bio-based solvent, no data is available in the Ecoinvent database and we resorted to published literature.[41]

Table S16. Selected midpoint results comparison of DFX production and several solvents (per kg unless specified).

**Scenario**	**Climate change**	**Terrestrial acidification**	**Freshwater eutrophication**	**Human toxicity**	**Freshwater ecotoxicity**	**Marine ecotoxicity**	**Terrestrial ecotoxicity**	**Natural land transformation**	**Water depletion**	**Fossil fuel depletion**
**Unit**	kg CO_2-eq	kg SO_2-eq	kg P-eq	kg 1,4-DCB-eq	kg 1,4-DCB-eq	kg 1,4-DCB-eq	kg 1,4-DCB-eq	m^2	m^3	kg oil-eq
DFX production										
Corn cob	-1.63	0	0	0	0	0	0	0	0	0
Xylose	-0.87	7.09E-03	5.33E-04	0.11	0.06	0.08	6.46	1.82	0.02	0.06
Sulfuric acid	0.13	1.04E-02	4.96E-05	1.64E-02	2.12E-02	3.01E-02	4.88	5.08E-03	2.02E-02	0.03
Sodium hydroxide	1.28	5.03E-03	5.55E-04	8.40E-02	7.02E-02	9.26E-02	6.21	3.20E-02	3.17E-02	0.30
Bio-based Methanol	0.28	1.18E-03	1.39E-04	3.29E-02	2.28E-02	3.08E-02	2.74	1.55E+00	1.14E-02	0.07
Bioethanol	0.42	7.33E-03	8.36E-05	1.87E-02	1.17E-02	1.51E-02	1.38	9.71E-01	8.97E-03	0.09
Paraformaldehyde	1.09	2.08E-03	1.85E-04	6.50E-02	4.34E-02	5.72E-02	4.26	1.32E-02	6.52E-03	0.82
HCl	0.57	3.50E-03	3.26E-04	5.75E-02	5.42E-02	7.10E-02	5.02	1.85E-02	1.70E-02	0.16
Ethyl acetate	3.49	1.12E-02	1.15E-03	1.73E-01	1.26E-01	1.66E-01	11.53	6.79E-02	5.16E-02	1.61
Diethyl ether	7.78	6.41E-02	4.21E-03	5.88E-01	1.13E+00	1.48E+00	132.12	1.83E-01	1.87E-01	2.72
1-butanol	3.95	1.29E-02	1.29E-03	1.73E-01	1.03E-01	1.38E-01	7.47	6.68E-02	2.81E-02	1.87
2-methyltetrahydrofuran	0.87	1.18	0.37	0.11	0.56	0.05	1.39E-03	0.07	0.03	4.06E-05
Di-n-Butylether	7.85	4.77E-02	2.91E-04	0.48	0.73	0.95	81.05	0.17	0.13	0.40
Utilities and wastes										
Steam (/MJ)	0.12	2.07E-04	1.24E-05	2.08E-03	6.49E-04	9.75E-04	0.16	9.13E-04	1.07E-04	0.03
Cooling (/MJ)	0.06	7.13E-05	6.41E-06	3.18E-03	3.94E-03	5.05E-03	0.32	3.28E-04	8.74E-04	0.02
Electricity (/kWh)	0.02	6.04E-05	5.07E-06	3.02E-03	5.02E-03	6.14E-03	0.26	1.31E-03	1.47E-02	1.77E-03
Tap water	0.00	1.13E-06	2.03E-07	2.60E-04	1.63E-05	2.25E-05	0.00	8.27E-06	3.46E-06	8.27E-05
Infrastructure	3.55E-03	1.81E-05	1.71E-06	1.36E-03	9.68E-04	1.27E-03	0.10	4.57E-04	2.46E-05	5.13E-04
Wastewater (/m^3)	8.41	5.67E-03	1.73E-02	9.12E-02	7.55E-02	9.81E-02	7.15	3.12E-02	5.18E-02	0.25
Salts	2.85E-04	1.87E-06	2.24E-04	5.05E-03	1.96E-01	2.42E-01	0.00	7.92E-04	4.17E-06	8.29E-05
Spent solvent	1.96	8.91E-04	2.78E-04	1.33E-02	0.00E+00	0.00E+00	0.00	4.66E-05	4.61E-03	0.06
Solvent comparison										
DMF	3.08	7.11E-03	8.94E-04	1.62E-01	1.26E-01	1.61E-01	11.19	4.74E-02	4.96E-02	1.56
DMSO	1.38	1.24E-02	3.27E-04	8.52E-02	6.77E-02	8.87E-02	6.09	1.95E-02	2.56E-02	0.86
NMP	6.78	1.91E-02	1.92E-03	3.20E-01	2.42E-01	3.21E-01	24.01	1.38E-01	2.74E-01	2.33
DMAc	4.10	1.06E-02	1.02E-03	1.98E-01	1.47E-01	1.90E-01	13.79	5.64E-02	6.29E-02	1.83
1,4-dioxane	4.91	1.29E-02	1.24E-03	2.09E-01	1.65E-01	2.17E-01	13.38	7.93E-02	1.50E-01	2.09
Ethylene Carbonate	1.71	4.27E-03	4.03E-04	8.57E-02	8.07E-02	1.05E-01	8.25	2.21E-02	1.44E-02	0.79
GVL	0.19	3.00E-03	7.20E-05	3.00E-03	9.10E-02	1.27E-01	1.20E-02	2.50E-01	4.60E-02	0.05

The colour assignment in the heat map in Figure 8, b was scaled using the standard score:

$$Standard\ score = \frac{x_{ij} - \mu_i}{\sigma_i}$$

where x_{ij} is the midpoint result in the ith category of solvent j; μ_i and σ_i are the mean and the standard deviation, respectively, of the results in the ith category among all solvents.

Table S17. LCIs for DFX production.

Flow	Xylose route	Corn cobs: Neutralized route	Corn cobs: Non-neutralized route
Xylose	1.090	0	0
Paraformaldehyde	0.359	0.154	0.147
Sulfuric acid	0.109	0	0
Sodium hydroxide	0.086	0.613	0
Tap water	0.001	2.320	0.001
2-methyltetrahydrofuran	0.004	0.052	0.002
Bioethanol	4.40E-04	0.006	0.008
Salt	0.169	0	0
Wastewater (m^3)	4.81E-04	0.004	0
Electricity	0.160	0.406	0.351
Cooling	1.250	4.393	0.095
Steam (MJ)	4.434	3.634	5.206
Corn cob	0	1.326	1.303
HCl	0	0.559	3.50E-04
Ethyl acetate	0	0	0
Di-n-Butylether	0	0	0.003
Spent solvent	0	0	0.466

Table S18. LCIs for corn cobs to xylose and for 2-methyltetrahydrofuran production (in kg unless otherwise specified).

Flow	per kg xylose	per kg 2-MeTHF
Inputs		
Corn cobs	2.676	
Steam from natural gas (MJ)	2.00E-04	
Electricity (kWh)	1.32E-04	0.4258
Cooling water (m^3)	0.134	7.806
Refrigeration -25ºC (MJ)	0.371	
Methanol (biobased)	0.757	
Sulfuric acid	0.340	0.823
Sodium hydroxide	0.273	
Tap water	4.146	4.840
Hydrogen		0.108
Bagasse (including heating)		15.461
Heating supplied by bagasse (MJ)		25.083
Outputs		
Residual pulp		10.568
gamma-valerolactone		0.069
Methanol		0.195
Spent solvent		0.018
Emissions to air		
Biogenic CO_2	2.463	
Water	1.197	
Emissions to water		
Water	4.343	29.496
Na_2SO_4	0.484	
NaOH	1.41E-04	
CO_2	0.028	

Table S19. Selected midpoint results for all considered scenarios (per kg DFX for xylose process, per kg of products otherwise).

Scenario	Climate change	Terrestrial acidification	Freshwater eutrophication	Human toxicity	Freshwater ecotoxicity	Marine ecotoxicity	Terrestrial ecotoxicity	Natural land transformation	Water depletion	Fossil fuel depletion
**Unit**	kg CO_2-eq	kg SO_2-eq	kg P-eq	kg 1,4-DCB-eq	kg 1,4-DCB-eq	kg 1,4-DCB-eq	kg 1,4-DCB-eq	m^2	m^3	kg oil-eq
**Xylose route**	0.164	0.015	0.002	0.164	0.130	0.168	10.912	2.001	0.037	0.565
**Corn cobs: neutralized route**	-0.115	0.068	0.020	0.125	0.133	0.138	9.511	0.047	0.042	0.608
**Corn cobs: non-neutralized route**	-0.386	0.004	0.001	0.031	0.019	0.024	2.206	0.018	0.009	0.334

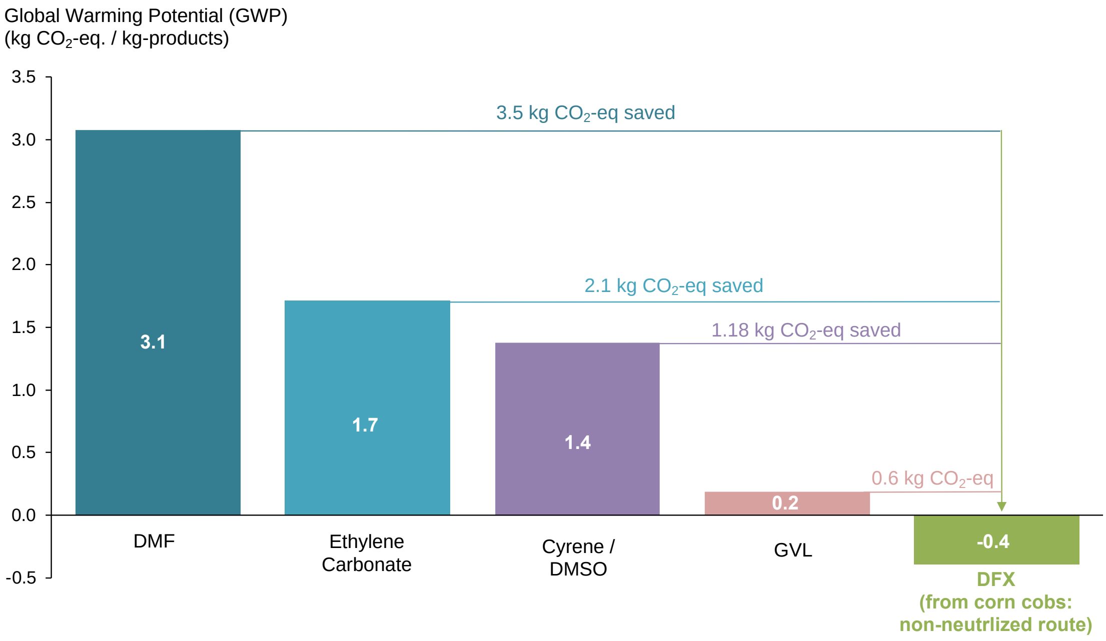

Figure S14. Comparison of the GWP impact for DFX produced from corn cobs by the non-neutralized route with other conventional bio-based solvents depicting potential CO_2-eq. savings.

S7. Biodegradability assessment by 301 F test

The principle of the 301F test is to monitor the oxygen consumption by microorganisms in closed respirometers (closed bottles with headspace) for 28 days as they break down the test compound.[4] During this process, CO_2 is released and then absorbed by concentrated NaOH placed under the measuring unit of the bottle, causing a negative overall pressure. As a result, the measuring heads record the pressure difference that can be converted to biochemical oxygen demand (BOD) and eventually to a percentage of the theoretical oxygen demand for the test compound (or biodegradation %) (see S7.2 below).

The mineral media used in the biodegradability test was prepared following the OECD 301F guidelines. Briefly, it consisted of 0.6 mM KH_2PO_4, 1.25 mM K_2HPO_4, 1.1 mM $Na_2HPO_4 \cdot 2H_2O$, 0.1 mM NH_4Cl, 0.2 mM $CaCl_2$, 92 µM $MgSO_4 \cdot 7H_2O$, 0.9 µM FeCl3·6H2O, and 2 mg/L N-allylthiourea as nitrification inhibitor. The resulting media had a pH of 7.4.

The inoculum was prepared by mixing the content of one capsule Polyseed (Seed Inoculum, Interlab, USA) with 500 ml of prepared mineral medium. The suspension was aerated for 1 h and then allowed to settle before use.

The test set-up consisted of OxiTop measuring system: vessels with pressure-measuring heads and rubber quivers filled with 3-4 NaOH pellets to absorb CO2. 164 ml of aerated mineral

media, containing inoculum was added to a test vessel with a magnetic stirrer. A test compound was added to the mixture to a final concentration of 100 mg/L as recommended by OECD. Filled vessels were placed in the test incubator set at 21°C with a shaking speed of 400 rpm. Biodegradation measurements were taken daily while refilling oxygen and NaOH pellets every 5 days. Each compound was tested in triplicate. The whole experiment was carried out in duplicate.

S7.1 Theoretical oxygen demand (ThOD)

Theoretical oxygen demand (ThOD) is the amount of oxygen required to oxidize a compound to its final oxidation products. For DFX full oxidation equation is the following:

$$C_7O_5H_{10} + 7\ O_2 = 7\ CO_2 + 5\ H_2O$$

Therefore, to fully oxidize 1mg of DFX (0.0057 mmol), 0.04 mmol of O_2 (or 1.286 mg) is required. For D-xylose, the calculated ThOD is 1.066 mg O_2/mg of the compound, respectively. The ThOD represents the maximum oxygen demand. The actual oxygen demand is then measured experimentally and is called the biochemical oxygen demand (BOD).

S7.2 Determination of biodegradation %

To convert the measured values into the BOD value, we multiplied the displayed digits by a factor of 10 (as recommended for the used sample volume of 164 ml). The following formula is the basis for BOD calculation using the digits from the OxiTop system:

$$BOD = \frac{M(O_2)}{R \cdot T_m} \cdot \left(\frac{V_t - V_l}{V_l} + \alpha \frac{T_m}{T_0} \right) \cdot \Delta p(O_2)$$

where $M(O_2)$ is the molecular weight of oxygen, R – gas constant, T_0 – reference temperature, T_m – measuring temperature, V_t – bottle volume, V_I – sample volume, α - Bunsen adsorption coefficient, $\Delta p(O_2)$ – difference of the oxygen partial pressure.

To calculate the biodegradation percentage, the amount of oxygen taken up by the bacteria in the test suspension was expressed as a percent of the ThOD:

$$Biodegradation\ (\%) = \frac{BOD\ test\ - BOD\ blank}{ThOD} * 100\%$$

Table S20. The gradation for biodegradability as defined by OECD.[31]

Status	Biodegradation
Readily biodegradable	≥ 60% within 10 days
Ultimately biodegradable	≥ 60% within 28 days
Inherently (primary) biodegradable	20-60% within 28 days
Non-biodegradable	≤ 20% in 28 days

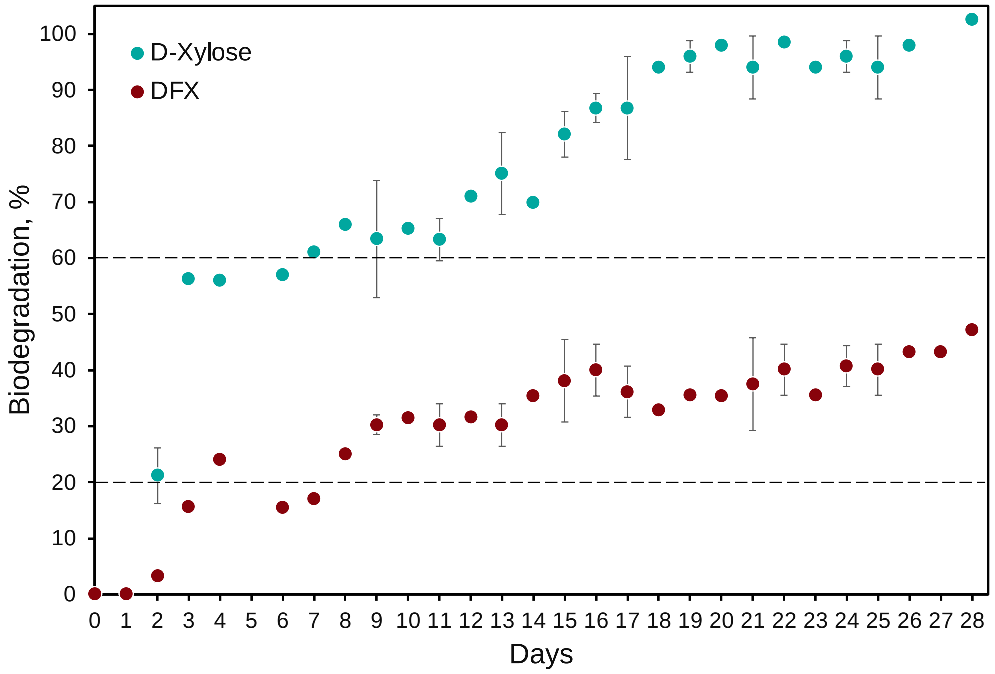

Figure S15. Biodegradation curve for D-xylose and DFX measured in accordance with 301F OECD guidelines within 28 days of incubation at 21°C. In the experiment, each data point was obtained in triplicate and then averaged. The error bars show the standard deviation between two values obtained from two independent experiments. Dashed lines show the window for the "inherently biodegradable" category. The biodegradation curve for DFX shows steady biodegradation behaviour without reaching a plateau phase within the standard 28-day test duration.

S7.3 Validation of the BOD results by alternative methods

To confirm the results obtained by manometric respirometry test 301F, we confirmed that DFX and xylose had been consumed through biodegradation as measured by BOD. To quantify DFX, we applied a GC-MS SICRIT (Soft Ionization by Chemical Reaction Interface Technology) procedure with increased sensitivity and plotted calibration curve first (Figure S16, a). The instrument was equipped with Zb-5ms column and each sample was injected three times in splitless mode with the temperature program from 35 to 350°C. Analysis of the sample from 28th incubation day showed concentration of DFX equal to 0.06 mg/ml corresponding to 54% of the initial DFX concentration (0.111 mg/ml) and, therefore, 46% degradation during the incubation. To quantify D-xylose, we used an HPLC equipped with an Aminex HPX-87H column at 60°C using 5 mM H_2SO_4 in water at a flow rate of 0.6 mL min^{-1} as the mobile phase. The calibration curve for D-xylose (Figure S16, b) was used to analyze the sample from 28th incubation day that showed 0.0003 mg/ml concentration of residual D-xylose corresponding to 0.3% of the initial xylose concentration (0.101 mg/ml) and, therefore, 99.7% degradation during the incubation.

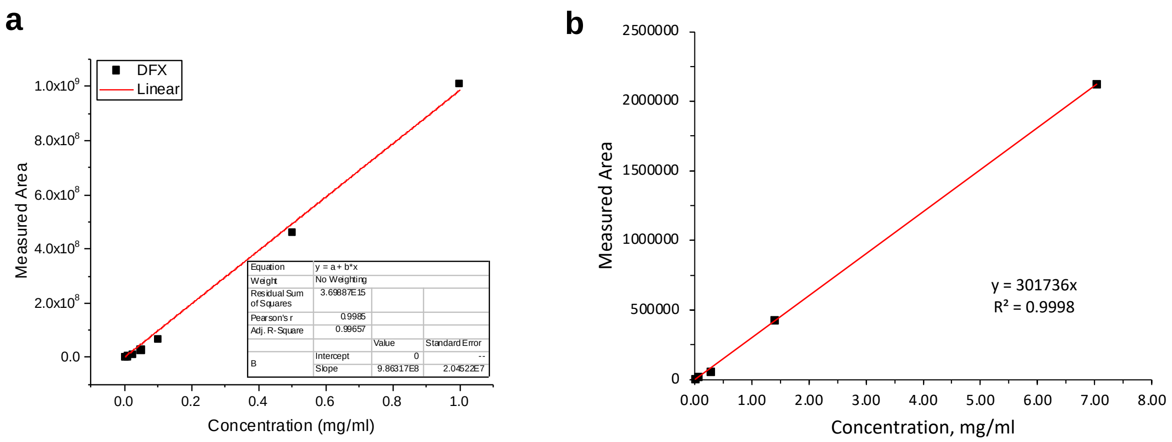

Figure S16. (a) Calibration curve for DFX measured by GC-MS SICRIT in a concentration range of 0.002-1 mg/mL. (b) Calibration curve for D-xylose measured by HPLC in a concentration range of 0.02-7 mg/mL.

References

(1) Constable, D. J. C.; Curzons, A. D.; Cunningham, V. L. Metrics to 'Green' Chemistry—Which Are the Best? *Green Chem.* **2002**, *4* (6), 521–527. https://doi.org/10.1039/B206169B.

(2) Iffland, K.; Sherwood, J.; Carus, M.; Raschka, A.; Farmer, T.; Clark, J. Calculation and Comparison of the " Biomass Utilization Efficiencies (BUE) " of Various Bio-Based Chemicals, Polymers and Fuels. *Nov. Inst* **2015**.

(3) Talebi Amiri, M.; Dick, G. R.; Questell-Santiago, Y. M.; Luterbacher, J. S. Fractionation of Lignocellulosic Biomass to Produce Uncondensed Aldehyde-Stabilized Lignin. *Nat. Protoc.* **2019**, *14* (3), 921–954. https://doi.org/10.1038/s41596-018-0121-7.

(4) OECD. *Test No. 301: Ready Biodegradability*; OECD Guidelines for the Testing of Chemicals, Section 3; OECD, 1992. https://doi.org/10.1787/9789264070349-en.

(5) Shuai, L.; Amiri, M. T.; Questell-Santiago, Y. M.; Héroguel, F.; Li, Y.; Kim, H.; Meilan, R.; Chapple, C.; Ralph, J.; Luterbacher, J. S. Formaldehyde Stabilization Facilitates Lignin Monomer Production during Biomass Depolymerization. *Science (80-.).* **2016**, *354* (6310), 329–333. https://doi.org/10.1126/science.aaf7810.

(6) Sicaire, A.-G.; Vian, M. A.; Filly, A.; Li, Y.; Bily, A.; Chemat, F. 2-Methyltetrahydrofuran: Main Properties, Production Processes, and Application in Extraction of Natural Products. In *Alternative Solvents for Natural Products Extraction*; 2014; pp 253–268. https://doi.org/10.1007/978-3-662-43628-8_12.

(7) Stephenson, R. M. Mutual Solubilities: Water-Ketones, Water-Ethers, and Water-Gasoline-Alcohols. *J. Chem. Eng. Data* **1992**, *37* (1), 80–95. https://doi.org/10.1021/je00005a024.

(8) Glass, M.; Aigner, M.; Viell, J.; Jupke, A.; Mitsos, A. Liquid-Liquid Equilibrium of 2-Methyltetrahydrofuran/Water over Wide Temperature Range: Measurements and Rigorous Regression. *Fluid Phase Equilib.* **2017**, *433*, 212–225. https://doi.org/10.1016/j.fluid.2016.11.004.

(9) Komarova, A. O.; Dick, G. R.; Luterbacher, J. S. Diformylxylose as a New Polar Aprotic Solvent Produced from Renewable Biomass. *Green Chem.* **2021**, *23* (13), 4790–4799. https://doi.org/10.1039/d1gc00641j.

(10) Turton, R.; Shaeiwitz, J. A.; Bhattacharyya, D.; Whiting, W. B. *Analysis, Synthesis, and Design of Chemical Processes*, 5th Editoi.; Prentice Hall PTR, 2018.

(11) Online Browsing Platform (OBP) ISO. ISO 14040:2006(En), Environmental Management — Life Cycle Assessment — Principles and Framework.

(12) U.S. Bureau of Labor Statistics. CPI Home https://www.bls.gov/cpi/ (accessed Dec 19, 2023).

(13) Ulrich, G. D. *A Guide to Chemical Engineering Process Design and Economics*, 1st Editio.; John Wiley & Sons, 1984.

(14) CSIMarket. Chemical Manufacturing Industry Management Effectiveness and Trends https://csimarket.com/Industry/industry_ManagementEffectiveness.php?ind=101&hist=12 (accessed Mar 8, 2024).

(15) Parra-Ramírez, D.; Martinez, A.; Cardona, C. A. Technical and Economic Potential Evaluation of the Strain Escherichia Coli MS04 in the Ethanol Production from Glucose and Xylose. *Biochem. Eng. J.* **2018**, *140*, 123–129. https://doi.org/10.1016/j.bej.2018.09.015.

(16) Mountraki, A. D.; Koutsospyros, K. R.; Mlayah, B. B.; Kokossis, A. C. Selection of Biorefinery Routes: The Case of Xylitol and Its Integration with an Organosolv Process. *Waste and Biomass Valorization* **2017**, *8* (7), 2283–2300. https://doi.org/10.1007/s12649-016-9814-8.

(17) Hong, J.; Zhou, J.; Hong, J. Environmental and Economic Impact of Furfuralcohol Production Using Corncob as a Raw Material. *Int. J. Life Cycle Assess.* **2015**, *20* (5), 623–631. https://doi.org/10.1007/s11367-015-0854-2.
(18) Bangalore Ashok, R. P.; Oinas, P.; Forssell, S. Techno-Economic Evaluation of a Biorefinery to Produce γ-Valerolactone (GVL), 2-Methyltetrahydrofuran (2-MTHF) and 5-Hydroxymethylfurfural (5-HMF) from Spruce. *Renew. Energy* **2022**, *190*, 396–407. https://doi.org/10.1016/j.renene.2022.03.128.
(19) Gönen, M.; Rodene, D. D.; Panda, S.; Akcil, A. Techno-Economic Analysis of Boric Acid Production from Colemanite Mineral and Sulfuric Acid. *Miner. Process. Extr. Metall. Rev.* **2022**, *43* (3), 402–410. https://doi.org/10.1080/08827508.2021.1893721.
(20) Moncada, J.; Gursel, I. V.; Worrell, E.; Ramírez, A. Production of 1,3-butadiene and E-caprolactam from C6 Sugars: Techno-economic Analysis. *Biofuels, Bioprod. Biorefining* **2018**, *12* (4), 600–623. https://doi.org/10.1002/bbb.1876.
(21) Aui, A.; Wang, Y.; Mba-Wright, M. Evaluating the Economic Feasibility of Cellulosic Ethanol: A Meta-Analysis of Techno-Economic Analysis Studies. *Renew. Sustain. Energy Rev.* **2021**, *145*, 111098. https://doi.org/10.1016/j.rser.2021.111098.
(22) ECHEMI. Market Price & Insight: Ethyl acetate https://www.echemi.com/productsInformation/pid_Seven5613-ethyl-acetate.html (accessed Jan 18, 2023).
(23) Marsden Jacob Associates. Estimate of the Cost of Hazardous Waste in Australia Report Prepared for the Department of the Environment. **2014**.
(24) Boulder County. Hazardous Waste Disposal Costs for Businesses https://bouldercounty.gov/environment/hazardous-waste/disposal-costs-for-businesses/ (accessed Jan 18, 2023).
(25) U.S. Energy Information Administration (EIA). Electricity power monthly https://www.eia.gov/electricity/ (accessed Jan 18, 2023).
(26) Camp, J. E.; Nyamini, S. B.; Scott, F. J. Cyrene^{TM} Is a Green Alternative to DMSO as a Solvent for Antibacterial Drug Discovery against ESKAPE Pathogens. *RSC Med. Chem.* **2020**, *11* (1), 111–117. https://doi.org/10.1039/C9MD00341J.
(27) Dastidar, R. G.; Kim, M. S.; Zhou, P.; Luo, Z.; Shi, C.; Barnett, K. J.; McClelland, D. J.; Chen, E. Y. X.; Van Lehn, R. C.; Huber, G. W. Catalytic Production of Tetrahydropyran (THP): A Biomass-Derived, Economically Competitive Solvent with Demonstrated Use in Plastic Dissolution. *Green Chem.* **2022**, *24* (23), 9101–9113. https://doi.org/10.1039/d2gc03475a.
(28) Wernet, G.; Bauer, C.; Steubing, B.; Reinhard, J.; Moreno-Ruiz, E.; Weidema, B. The Ecoinvent Database Version 3 (Part I): Overview and Methodology. *Int. J. Life Cycle Assess.* **2016**, *21* (9), 1218–1230. https://doi.org/10.1007/s11367-016-1087-8.
(29) Mutel, C. Brightway: An Open Source Framework for Life Cycle Assessment. *J. Open Source Softw.* **2017**, *2* (12), 236. https://doi.org/10.21105/joss.00236.
(30) Goedkoop, M.; Heijungs, R.; Huijbregts, M.; Schryver, A. De; Jaap Struijs; Zelm, R. van. *ReCiPe 2008: A Life Cycle Impact Assessment Method Which Comprises Harmonised Category Indicators at the Midpoint and the Endpoint Level*; RIVM, Bilthoven, NL, 2016.
(31) Sakuth, M.; Mensing, T.; Schuler, J.; Heitmann, W.; Strehlke, G.; Mayer, D. Ethers, Aliphatic. *Ullmann's Encyclopedia of Industrial Chemistry*; Wiley, 2010. https://doi.org/10.1002/14356007.a10_023.pub2.
(32) Khoo, H. H.; Wong, L. L.; Tan, J.; Isoni, V.; Sharratt, P. Synthesis of 2-Methyl Tetrahydrofuran from Various Lignocellulosic Feedstocks: Sustainability Assessment via LCA. *Resour. Conserv. Recycl.* **2015**, *95*, 174–182. https://doi.org/10.1016/j.resconrec.2014.12.013.

(33) Anukam, A. I.; Goso, B. P.; Okoh, O. O.; Mamphweli, S. N. Studies on Characterization of Corn Cob for Application in a Gasification Process for Energy Production. *J. Chem.* **2017**, *2017*, 1–9. https://doi.org/10.1155/2017/6478389.
(34) Manker, L. P.; Dick, G. R.; Demongeot, A.; Hedou, M. A.; Rayroud, C.; Rambert, T.; Jones, M. J.; Sulaeva, I.; Vieli, M.; Leterrier, Y.; Potthast, A.; Maréchal, F.; Michaud, V.; Klok, H. A.; Luterbacher, J. S. Sustainable Polyesters via Direct Functionalization of Lignocellulosic Sugars. *Nat. Chem.* **2022**, *14* (9), 976–984. https://doi.org/10.1038/s41557-022-00974-5.
(35) Jaffe, G.; Szkrybalo, W.; Weinert, P. Process for Producing Xylose. US3784408A, 1974.
(36) Slater, C. S.; Savelski, M. J.; Hitchcock, D.; Cavanagh, E. J. Environmental Analysis of the Life Cycle Emissions of 2-Methyl Tetrahydrofuran Solvent Manufactured from Renewable Resources. *J. Environ. Sci. Heal. Part A* **2016**, *51* (6), 487–494. https://doi.org/10.1080/10934529.2015.1128719.
(37) Ntimbani, R. N.; Farzad, S.; Görgens, J. F. Techno-economics of One-stage and Two-stage Furfural Production Integrated with Ethanol Co-production from Sugarcane Lignocelluloses. *Biofuels, Bioprod. Biorefining* **2021**, *15* (6), 1900–1911. https://doi.org/10.1002/bbb.2289.
(38) Leal Silva, J. F.; Mariano, A. P.; Maciel Filho, R. Economic Potential of 2-Methyltetrahydrofuran (MTHF) and Ethyl Levulinate (EL) Produced from Hemicelluloses-Derived Furfural. *Biomass and Bioenergy* **2018**, *119*, 492–502. https://doi.org/10.1016/j.biombioe.2018.10.008.
(39) M. de O. Camargo, J.; Marcela Gallego Ríos, J.; C. Antonio, G.; T.C. Leite, J. Physicochemical Properties of Sugarcane Industry Residues Aiming at Their Use in Energy Processes. In *Sugarcane - Biotechnology for Biofuels*; IntechOpen, 2021. https://doi.org/10.5772/intechopen.95936.
(40) Siemens Global Website. Energy generated from biomass https://www.siemens-energy.com/global/en/offerings/renewable-energy/biomass-to-power.html (accessed Jul 8, 2023).
(41) Han, J.; Son, M.; Kang, D. Process Design and Environmental Analysis for Catalytic Production of Gamma-Valerolactone from Kenaf. *J. Ind. Eng. Chem.* **2023**, *120*, 254–260. https://doi.org/10.1016/j.jiec.2022.12.032.
